# Novel Small-Molecule Treatment and Emerging Biological Therapy for Psoriasis

**DOI:** 10.3390/biomedicines13040781

**Published:** 2025-03-23

**Authors:** Yuanyuan Li, Yiheng Cheng, Yuchen Cai, Zhenduo Duan, Hong Xu, Yunan Huang, Xiaonan Ma, Xiaofei Xin, Lifang Yin

**Affiliations:** 1 Department of Pharmaceutics, China Pharmaceutical University, Nanjing 210009, China; lucian0310@163.com (Y.L.); kevincheng@stu.cpu.edu.cn (Y.C.); caiyc0125@foxmail.com (Y.C.); dzdd1stu@163.com (Z.D.); xhky2004@outlook.com (H.X.); 15950531867@139.com (Y.H.); 2 The Public Laboratory Platform of China Pharmaceutical University, Nanjing 210009, China; maxiaonan512@126.com; 3 NMPA Key Laboratory for Research and Evaluation of Pharmaceutical Preparations and Excipients, China Pharmaceutical University, Nanjing 210009, China; 4 Key Laboratory of Drug Quality Control and Pharmacovigilance, China Pharmaceutical University, Nanjing 210009, China; 5 State Key Laboratory of Natural Medicine, China Pharmaceutical University, Nanjing 210009, China

**Keywords:** small molecule, biomacromolecule, cell-based therapy, drug delivery system, psoriasis

## Abstract

Psoriasis is an immune-related disorder that is marked by abnormal thickening of the skin, the rapid multiplication of keratinocytes, and complex interactions between immune cells and the affected areas. Although psoriasis cannot currently be cured, drugs can alleviate symptoms by regulating immune homeostasis and preventing comorbidities. There are many types of drugs to treat psoriasis: small-molecule drugs, including corticosteroids; retinoids; vitamin D analogs; and immunosuppressants, such as glucocorticoid ointment, tretinoin cream, methotrexate tablets, etc. Macromolecular biological drugs, such as Certolizumab, Secukinumab, Guselkumab, etc., include monoclonal antibodies that target various inflammatory signaling pathways. Compared with traditional small-molecule drugs, biological therapies offer better targeting and lower systemic side effects, but their high costs and invasive administration modes constrict their widespread use. Spesolimab is the latest biological agent used to target the interleukin-36 receptor (IL-36R) to be approved for market use, which significantly reduces the risk of general pustular psoriasis (GPP) flare by 84%. Additionally, there are several biological agents used to target the interleukin-23/T helper 17 cell pathway that have already entered Phase II and III clinical trials. At present, the first-line therapeutic strategy for mild psoriasis is topical administration. Systemic therapy and phototherapy are preferred for treating moderate to severe types. However, the current therapeutic drugs for psoriasis cannot completely meet the clinical needs. More advanced drug delivery systems with optimized target effects and better bioavailability are required. Nanocarriers are emerging for the delivery of proteins, nucleic acids, and cell-based therapies. In this review, we analyze the current status of psoriasis therapeutics and discuss novel delivery systems for diverse psoriasis drugs, as well as emerging cell-based therapies. We also summarize the therapeutic effectiveness of different delivery strategies.

## 1. Introduction

Psoriasis, marked by clear-edged, reddish plaques covered with shiny, silvery-white scales, is an autoimmune skin disease typically appearing on the elbows, knees, scalp, back, and nails [1]. Psoriasis affects about 125 million people globally, with regional prevalence variations affecting 0.14% of the population in East Asia at its low end and 1.99% in Australasia at its high end [2]. There is no gender distinction in its epidemiology in most regions. Its prevalence and incidence are lower in children than in adults [3]. The peak incidence generally appears at ages 35–44 and 50–54 [4]. In 2024, the worldwide market for psoriasis therapies reached a valuation of USD 31.61 billion. The compound annual growth rate is estimated as 8% for the decade spanning 2024 to 2034, indicating that the market will surge to USD 68.24 billion in 2034 [5]. The disease manifests in five primary forms: plaque, guttate (or eruptive), inverse, pustular, and erythrodermic. Psoriasis arises from the intricate interaction of genetic and environmental influences, particularly in individuals carrying the HLA-C*06:02 risk allele. External stimulations, such as wounds, stress, smoking, poor dietary habits, certain medications, and alcohol intake, also play a significant role [6]. The HLA-C*06:02 allele has been identified by studies as a high-risk gene that participates in psoriasis pathogenesis. This allele encodes the major histocompatibility complex class I (MHC I), enabling the presentation of autoantigens to T cells [7] and melanocytes [8] and enhancing autoimmune responses, which contribute to the pathogenesis of psoriasis [9]. External factors, such as trauma, infection, or certain medications, can trigger the secretion of self-nucleotides. These biomacromolecules combine with antimicrobial peptides produced in an inflammatory situation to form complexes. These molecular complexes subsequently engage with plasmacytoid dendritic cells (pDCs) and membrane receptor Toll-like receptors (TLRs), stimulating the secretion of interferon-α (IFN-α) and interferon-β (IFN-β). The released interferons activate myeloid dendritic cells (mDCs), which subsequently produce proinflammatory cytokines, including interleukin-12 and -23 and tumor necrosis factor (TNF). IL-23 interacts with receptors, promoting naive T cells’ transformation into specialized T helper cells 1 and 17. Once activated, TH1 cells release TNF-α; TH17 and TH22 cells produce IL-17A, IL-17F, and IL-22. TNF-α attracts more immune cells by exacerbating inflammatory responses, leading to their accumulation in affected skin areas. IL-17A and F activate keratinocytes to secret antimicrobial peptides, chemokines, and other inflammatory mediators, further exacerbating inflammation. IL-22 promotes keratinocyte proliferation and disrupts the skin barrier function. These signaling molecules trigger the excessive growth of keratinocytes, elevate the production of angiogenesis-promoting factors, and enhance the expression of adhesion molecules and the production of more proinflammatory molecules, creating a self-sustaining inflammatory loop [10].

The approach to managing psoriasis is tailored based on the severity of symptoms. For mild cases, local treatments are considered the most effective option [11]. Corticosteroids, analogs of vitamin D3, inhibitors of calcineurin, retinoids, and immunosuppressants, which are primarily small-molecule drugs, are used for treatment [12]. The drugs typically prescribed for psoriasis treatment include clobetasol propionate, betamethasone, calcipotriol, tazarotene, tretinoin, and methotrexate. These medications can be used either individually or in combination, administered through various topical formulations, such as creams, gels, pastes, lotions, ointments, and sprays [13]. With the development of technology, a diversity of nanocarriers have been designed to rectify the defects of traditional approaches regarding low bioavailability, systemic side effects, frequent dosing requirements, etc. [14]. Moreover, novel drug delivery systems have the potential to incorporate novel molecules into treatments for psoriasis [15].

There remains a need for precise therapeutic strategies with fewer side effects and improved compliance. In recent years, advancements in biomaterial-based therapies have opened new avenues for more effective topical treatments for psoriasis, offering promising prospects. Monoclonal antibodies and fusion proteins were widely used for the treatment of immune-mediated diseases [16]. Biologics with specific cytokines, including Tnf-α, IL-17, or IL-23, can provide better efficacy and safety than traditional treatment strategies and have been approved by the FDA for treating moderate to severe psoriasis [17]. In recent years, RNA interference (RNAi)-based targeted psoriasis therapy has been a promising therapy for psoriasis [18]. Ongoing scientific advancements are expected to yield more efficient and safer siRNA-based medications, which could significantly enhance psoriasis patients’ compliance and transform the landscape of psoriasis treatment [19].

Nevertheless, more severe psoriasis patients are not responding to existing therapeutic options. In such scenarios, cellular therapies, such as mesenchymal stem cells (MSCs), their extracellular vesicles [20], and regulatory T cells (Tregs) [21], have been explored. Notably, MSCs and their derived exosomes (MSC-EXO) exhibit significant immunoregulatory and anti-inflammatory effects [22]. Tregs are indispensable for maintaining immune balance and participating in preventing autoimmune disorders by inhibiting excessive immune reactions. Both offer promising prospects for psoriasis treatment. This review summarizes the drug delivery strategies of therapeutics, including small molecules and biologics, in recent years. Various drug delivery systems are described, along with their potent efficacy for psoriasis treatment. We also discussed the potential and current status of clinical translation for novel drug delivery systems [23].

## 2. Small Molecules

### 2.1. Corticosteroids

#### 2.1.1. Micro/Nanocarrier-Loaded Hydrogels

Corticosteroids are the primary treatment for all severity levels of psoriasis, used either alone or alongside systemic therapies. They exert their therapeutic effects via nuclear receptor interactions and rapid non-receptor-mediated mechanisms. The nuclear receptor interactions involve glucocorticoid receptors (GRs) activated by cortisol, leading to receptor homodimerization and binding to glucocorticoid response elements (GREs). In the absence of ligands, GRs remain in the cytoplasm, bound to proteins. Ligand binding disrupts this complex, allowing GRs to translocate to the nucleus. Upon dimerization and binding to GREs, GRs promote the transcription of genes that secrete anti-inflammatory cytokines. In contrast, rapid non-receptor-mediated mechanisms involve combining with membrane receptors, which mediates rapid glucocorticoid effects within minutes by second messengers. This pathway does not rely on protein synthesis but modulates the activity of monocytes, T cells, and platelets [24,25,26]. Corticosteroids are formulated in various preparations, especially ointments and creams. The choice of excipient can significantly influence the therapeutic and adverse effects by altering the pharmacokinetics of the corticosteroid. Consequently, the development of advanced and innovative delivery systems for corticosteroids is a major focus in pharmaceutical research [27].

**Figure 1:**
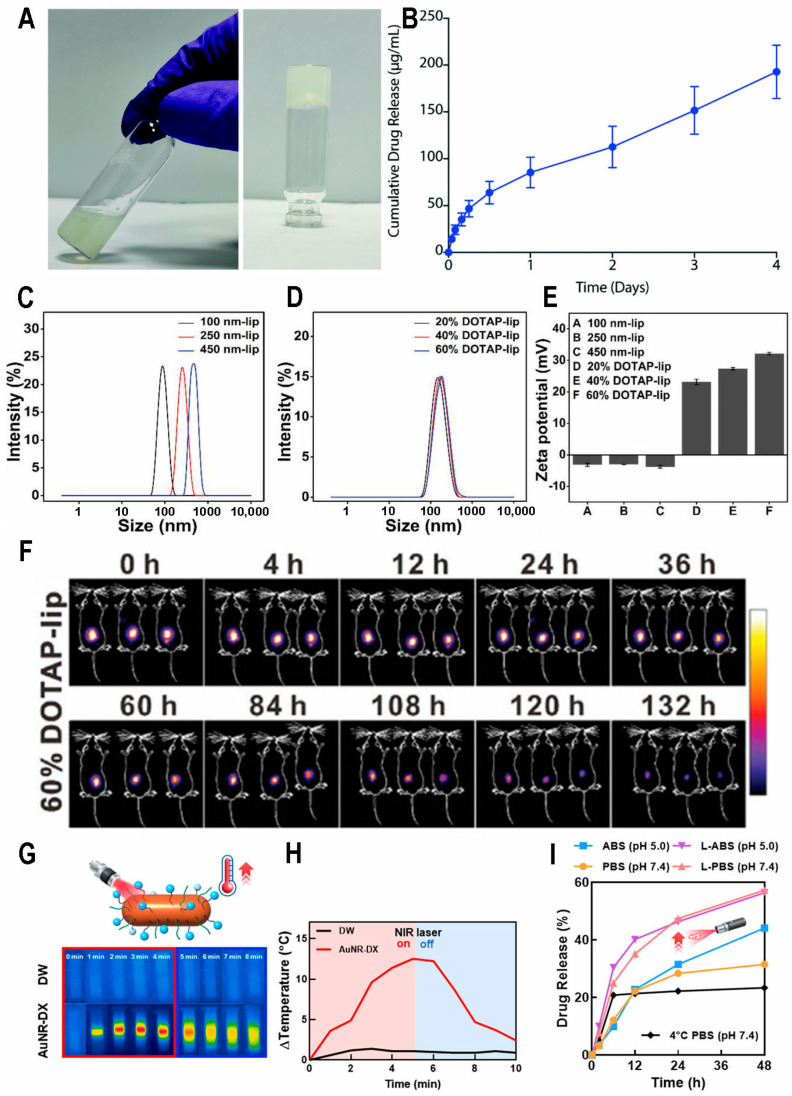
(**A**) Solution to the gel phase transition of B-gel. (**B**) In vitro release profile of betamethasone loaded in B-gel. Reproduced with permission from Ref. [31]. (**C**) The size distribution of different liposomes. (**D**) The size distribution of different DOTAP content liposomes. (**E**) The Zeta potential of different liposomes. (**F**) In vivo NIR-II fluorescence imaging of 60% DOTAP-lip. From black to white, the color bar reflects the intensity of fluorescence from weak to strong. Reproduced with permission from Ref. [34]. (**G**) The thermal imaging shows that AuNR-DX was photoactivated using near-infrared lasers (excitation wave: 808 nm). From blue to red, the temperature reflects from low to high. (**H**) The quantitative results of temperature variation in laser-activated nanorods. (**I**) Photoactivation promoted the Dex release in simulated cellular conditions. Reproduced with permission from Ref. [35].

At present, the main dosage form of corticosteroids on the market is ointment. Compared with ointment, hydrogel is aqueous; it leaves no residue, provides good moisturization, and has a non-greasy texture, making it favorable. Hydrogels can also maintain skin hydration, prevent itching, and provide a prolonged drug contact time. Additionally, hydrogels can be combined with liposomes, vesicles, solid lipid nanoparticles, and nanocarriers to enhance skin retention, penetration, and release profiles of drugs [28]. Jiang et al. developed a temperature-sensitive polymer-based dexamethasone (Dex) prodrug named ProGel-Dex. The dexamethasone molecule is connected to the HPMA molecule via a hydrazone bond, which endows ProGel-Dex with the ability to cleave under acidic conditions and activate at the lesion site. Its phase transforms from liquid at 4 °C to gel above 30 °C. Experimental results showed that the low-dose administration group (L group) could reduce the PASI score (Psoriasis Area and Severity Index) to below 2, which is significantly lower compared with the score of 5 observed in the imiquimod (IMQ)-induced model group. Histological hematoxylin–eosin staining (H&E staining) results revealed a reduction in epidermal thickness in the low-dose group. Enzyme-linked immunosorbent assay (ELISA) measurements of serum inflammatory factors indicated that the L group reduced IL-6 levels from 20 pg/mL to 0 pg/mL. The efficacy of ProGel-Dex in alleviating skin inflammation can be attributed to the reduction in serum IL-6 levels [29]. Kondiah et al. prepared hydrogenated cortisol-loaded sorbitan monostearate–polycaprolactone nanoparticles (HCT-SMS-PCL), which were encapsulated in a temperature-sensitive gel composed of thermosensitive carboxymethyl cellulose (CMC). The encapsulation efficiency (EE) of nanoparticles was 76%. The tube inversion method results indicated a state transition temperature of 33 ± 2 °C, suggesting its capability for drug delivery at physiological temperatures. The release rates of nanoparticles and hydrogel at pH 5.9 were 83.9% and 81.9%, indicating favorable drug release properties [30]. Traditional topical corticosteroid formulations face limitations, including inefficient drug encapsulation, outburst release, limited skin penetration, and potential toxicity concerns. Here, Rana et al. made a soft hydrogel (B-gel) for the localized delivery of betamethasone by conjugating glycine dipeptide with hydrophilic cholic acid. The preparation was under heating conditions and formed a gel upon cooling to room temperature (Figure 1A). About 50% of the betamethasone was released within one day in the release experiment, with no significant outburst release observed. The Franz cell diffusion shows >95% betamethasone skin permeation after 1 day (Figure 1B). In the IMQ-induced mouse model (ear), after one treatment cycle, B-gel reduced the overall PASI score from 12 to 2, which is comparable to the reduction achieved by the clinically used betamethasone valerate cream, which also lowered the PASI score from 12 to 2. Flow cytometry analysis of ear skin tissue samples revealed that B-gel achieves a 2.5-fold decrease in leukocyte (CD45+ cell) infiltration in comparison with untreated samples. This result was further corroborated using immunofluorescence staining [31]. Devi et al. reported a microsponge that was designed as the carrier of clobetasol propionate (CP) and encapsulated into carbomer gel. The results of drug release experiments revealed that the microsponge-loaded gel achieved sustained release for up to 9 h, with the release behavior following zero-order kinetics. This significantly minimizes the side effects and skin irritation associated with the burst release of CP [32]. Pradhan et al. combined fluocinonide acetate (FA) with nano-lipid carriers (NLCs). They embedded the NLCs in a gel containing salicylic acid (FSG) and prepared a gel containing both plain FA and salicylic acid (PFSG) as a control. The findings indicated that FSG exhibited a significantly prolonged release duration compared with PFSG. Specifically, the FA release period for FSG was extended beyond 24 h, whereas the PFSG formulation achieved over 90% FA release in less than 7 h. According to the quantitative results, in the stratum corneum, the FA content in the FSG group was 1, while in the PFSG group, it was 0.5. In the viable layer, the FSG group was 1.7, compared with 0.6 in the PFSG group (μg/0.64 cm^2^). The results indicate that NLCs enabled FA to penetrate the skin better. The ELISA results of skin samples showed that, compared with the positive control (IMQ-induced mouse), the levels of TNF-α in the FSG and PFSG groups decreased by 1.81- and 1.2-fold, respectively. The concentrations of IL-17 were reduced by 1.84- and 1.14-fold, while IL-22 levels decreased by 2.1- and 1.3-fold, respectively. These results suggest that NLC loading enhanced the local delivery efficiency of FA and salicylic acid [33].

Iontophoresis can improve local drug permeation through the *Stratum corneum* [36]. A dual-drug co-release patch, powered by an integrated magnesium battery, employs a hydrogel with an ionic hyperbranched poly (amidoamine) dendrimer, serving as both the cathode and drug reservoir. This patch can hold the release of dexamethasone (Dex) and tannic acid (Ta) in a controlled way, overcoming the limitation of a single treatment and the risk of sudden release. When a 1 kΩ resistor was loaded, the cumulation of drug release was 44.3% of Ta and 40.5% of Dex. Both drugs were released as intended, and the inflammatory skin was restored after a 5-day administration [37]. Shah et al. invented a desoximetasone-loaded niosome (non-ionic surfactant vesicles) that improves the solubility of desoximetasone and incorporated stearic acid into niosomes, which slowed the drug release from the interior. Niosome was loaded into carbomer 980 gel as the test gel, while Topicort^®^ was used as the reference gel. In vitro drug release experiments demonstrated that this formulation released approximately 5% of the drug within 5 h compared with the 15% drug release presented in the reference formulation. A drug study was conducted to evaluate the retention of the test gel and reference gel. The results revealed that the 30.88 ng corticosteroid test gel was retained in the skin per milligram compared with 26.01 ng/mg for the reference product [38]. Kumar et al. prepared a nano-sponge with polymer β-cyclodextrin and cross-linker diphenyl carbonate, which improved the solubility of clobetasol propionate (CP) by 45-fold. CP-loaded cyclodextrin nano-sponge (CP-CDNS) was embedded into carbomer hydrogel and applied to the psoriasis mouse model. It significantly amplified the degree of orthokeratosis by 69.44 ± 0.62% and reduced the relative epidermal thickness to 31.66 ± 8.96% compared with the untreated group [39].

#### 2.1.2. Microneedles

Microneedles (MNs) are miniature needles that typically measure between tens and thousands of micrometers in length. MNs are engineered to physically penetrate the outer barrier (stratum corneum), thereby providing the channel for drug delivery. Compared with traditional hypodermic injections, MNs provide a minimally invasive transdermal delivery method, making them a promising option, particularly for managing chronic conditions. Their versatility in material selection and design enables precise dosage control and flexibility in drug delivery. MNs facilitate targeted drug release, enhance biodistribution, and minimize drug loss caused by systemic circulation, degradation, clearance, or tissue diffusion. They hold significant potential for treating various skin disorders, including acne, atopic dermatitis, psoriasis, and skin cancer, by enabling targeted immunomodulator delivery to skin-resident immune cells [40].

Recently, Qu et al. used microneedles as the reservoir for liposomes. Their study examined how the liposomes’ physiochemical properties (such as size and potential) (Figure 1C–E) influence skin retention and cell internalization. Their findings revealed that 100 nm anionic liposomes achieved higher cellular absorption, whereas larger anionic liposomes (200 nm and 450 nm) improved skin retention and extended drug release within the body. Additionally, converting the surface charge of 250 nm anionic liposomes into cationic liposomes can further improve cell uptake. Indocyanine green-loaded liposomes revealed that cationic liposomes can prolong the fluorescence signal in the skin by 132 h (Figure 1F). Finally, dexamethasone was encapsulated in optimized liposomes for administration. Compared with the model group, symptoms were significantly relieved, and epidermal thickening was reduced [34]. However, most water-soluble microneedles have difficulty in effectively trapping hydrophobic glucocorticoids. Here, Wang et al. utilized hydroxypropyl-β-cyclodextrin to form a water-soluble inclusion complex with triamcinolone acetonide, and this complex was used to construct a supramolecular microneedle (TAMN) with low shrinkage and rapid dissolution properties. The microneedles effectively penetrated the thickened outer skin layer of IMQ-treated mice, achieving a depth of 300 μm, and successfully delivered triamcinolone acetonide to the targeted region. Histopathological analysis revealed that the use of TAMN reduced the epidermal thickness of imiquimod-treated ears from 111 ± 5.59 μm to 34.53 ± 0.87 μm. Additionally, it significantly decreased the expression of Ki67, which is indicative of keratinocyte proliferation and reflects the severity of psoriasis [41]. Wang et al. designed a multi-functional structured color triboelectric microneedle patch (SMP) made from budesonide-coated polyacrylamide–polyethylene glycol diacrylate–lithium chloride (PAM-PEGDA-LiCl) ionic hydrogel with an anti-opal microneedle structure. The release of budesonide is driven by the electric charge generated from the friction between the ionic hydrogel and the skin, known as a triboelectric nanogenerator (TENG). This mechanism enables controlled drug release through continuous self-powered stimulation [42].

#### 2.1.3. Nanoparticles

Nanoparticles have aroused researchers’ interest due to their small size and convenience of preparation. Loading corticosteroids onto nanoparticles can result in good penetration and retention effects after administration. Dexamethasone can cause a series of side effects due to its limited skin permeability. Gold nanorods have less toxicity and better biocompatibility, can absorb NIR to convert energy into heat energy (Figure 1G,H), and promote drug release (Figure 1I) compared with other inorganic materials. Dexamethasone was coupled with gold nanorods (AuNR-DX) to facilitate skin permeation and improve the psoriasis treatment effect. By measuring the inflammatory factors (IL-6, TNF-α, and CCL17) in keratinocytes, the combination of AuNR-DX and 308 nm excimer laser demonstrated excellent efficacy, with a dosage that is 1/5 of that when DX is used alone. Such a low dosage implies a reduced risk of systemic side effects [35]. Skin penetration of fluocinolone acetonide was enhanced by combining fluocinolone acetonide with a biodegradable polyglutamate carrier named PGA-FLUO. PGA-FLUO was loaded with hyaluronic acid–polyglutamate cross-polymer. It was found that the formulation decreased IL-6 levels by around 20% in comparison with the untreated control. Moreover, few conjugates were present in the dermis, which reduces the possibility of fluorine acetate easily entering the systemic circulation and causing side effects [43]. The stratum corneum at the focal site of psoriasis is more incomplete than that in healthy skin. Using this scenario, Mai et al. prepared a bio-adhesive nanoparticle (BNP) modified by tris(hydroxymethyl)aminomethane (Tris-BNP). When the nanoparticle spreads and penetrates into the epidermis, the aldehyde group of BNPs exposed by tris(hydroxymethyl)aminomethane molecules binds to the amine group in the diseased skin, which can prolong the preservation of nanoparticles in psoriasis skin. Tris-BNPs exhibited penetration depths of 414.5 ± 100.0 μm in human psoriatic skin and 128.3 ± 57.1 μm in mouse psoriatic skin. Research has shown that the thickness of the stratum corneum and epidermis in psoriasis mice is approximately 7 μm and 200 μm, while in psoriatic patients, these values are approximately 90 μm and 500 μm. These findings confirm that Tris-BNPs are retained within the epidermal layer following topical administration on psoriatic skin. Tris-BNPs can maintain the local drug concentration in the therapeutic window for 3 days and are not susceptible to external conditions such as sweating, moderation, and active wiping due to their high retention [44].

#### 2.1.4. Cubosomes

Cubosomes are distinct nanoscale vesicular systems made up of biodegradable lipids and water, which organize into a bicontinuous cubic liquid crystalline structure. They possess an exceptional capacity to solubilize and encapsulate molecules with varying properties, including hydrophilic, hydrophobic, and amphiphilic compounds, alongside showcasing excellent biocompatibility and strong bio-adhesive characteristics. The researchers loaded powerful corticosteroid betamethasone dipropionate (BD) and salicylic acid (SA) into cubosomes and modified the adhesive and rheological properties of cubosomes by incorporating sodium carboxymethyl cellulose, thereby extending the contact time of the formulation with the scalp. The change in ear thickness of psoriatic mice treated with BD/SA cubosomes was 11 × 10^−2^ mm, while that of the commercial BD/SA lotion was 17 × 10^−2^ mm, which indicates that BD/SA cubosomes exhibit superior anti-psoriatic efficacy [45].

### 2.2. Immunosuppressants

At present, the main methotrexate preparations on the market for psoriasis therapy are mainly oral dosage forms, but their low bioavailability and large systemic toxicity limit their application. Researchers have changed the delivery route of methotrexate to topical preparations to avoid system side effects and improve bioavailability [46].

#### 2.2.1. Hydrogel

**Figure 2:**
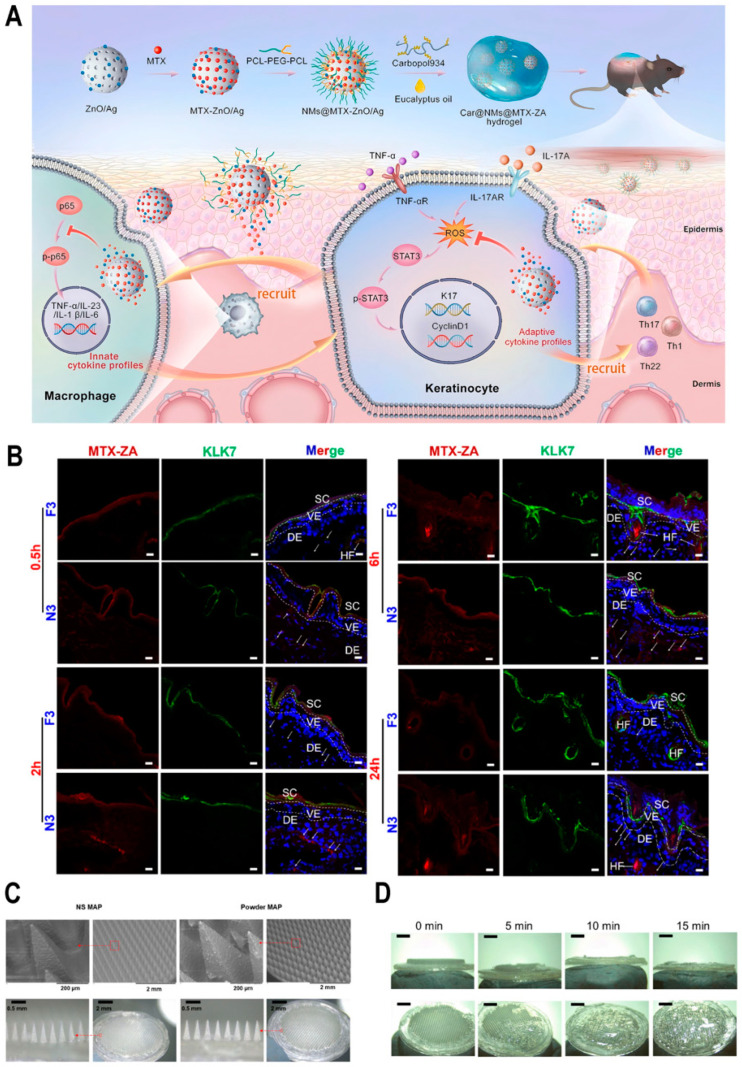
(**A**) The construction process and therapeutic mechanisms of MTX-ZA hydrogel. (**B**) The CLSM imaging shows the skin penetration of MTX-ZA at 0.5 h, 2 h, 6 h, and 24 h. MTX-ZA was labeled using Nile red (red color). Cytokeratin 17 and Kallikrein 7 (KLK7) antibodies (green) were used to distinguish the stratum corneum (SC) layer, hair follicles (HFs), visible epidermal (VE), and dermis (DE). The nuclei were dyed with DAPI (blue). White arrows indicate the Nile red-labeled MTX-ZA. Scale bar: 50 nm. Reproduced from Ref. [50]. (**C**) NS MAP and powder MAP morphology via SEM (scanning electron microscope) and digital image. The red arrows and boxes indicate the partially enlarged figures of the microstructure of microneedles (**D**). The skin dissolution state of NS MAPs at 0 min, 5 min, 10 min, and 15 min. Scale bar: 2 mm. Reproduced with permission from Ref. [51].

Kochkina et al. proposed an external water gel based on iota-carrageenan (iCR). β-cyclodextrin (β-CD) was incorporated into carrageenan gel to form a clathrate with MTX, which significantly improved the solubility of MTX in water. The addition of β-CD accelerates the release of MTX, achieving 100 wt% within 4 h, whereas, without β-CD, the cumulative release is only 35 wt% over the same period. Transdermal transport experiments reveal that the presence of β-CD reduces the transport efficiency by 10% within 5 h. The inclusion of β-CD facilitates the rapid release of MTX while limiting its entry into the systemic circulation, thereby retaining a significant amount of MTX at the psoriatic lesion site and enabling long-lasting therapeutic effects [47]. Photodynamic therapy (PDT) has long been accepted as a noninvasive and cosmetically favorable treatment for psoriasis. ALA (5-aminolevulinic acid), as a photosensitizer for PDT, has been approved by the FDA. Externally administered ALA is converted inside cells into the photosensitive compound protoporphyrin IX (PpIX). When exposed to light, the accumulated PpIX produces reactive oxygen species (ROS) that trigger cell transition to apoptosis and necrosis in the targeted tissue, which aligns with the therapeutic goal of inhibiting cell proliferation in psoriatic lesions. Due to the complexity of psoriasis pathology, it often exhibits insufficient responsiveness to monotherapy. To achieve better therapeutic outcomes, drug–photodynamic combination therapy has gained increasing attention from researchers. To overcome the skin barrier in psoriasis, Wang et al. created a nanogel by combining chitosan and hyaluronic acid for the topical administration of MTX and ALA. While the compound suspension only penetrated 20 μm into the epidermis, the nanogel achieved a deeper penetration of 70 μm. The apoptosis rates for LPS-induced HaCaT cells were 50.2% with the suspension and 78.6% with the nanogel. Additionally, nanogel reduced TEWL (transepidermal water loss) values to 16.20 ± 0.98 g/m^2^·h [48]. A zinc nanospheres were reported previously by Zhang et al. [49]. Xu et al. reported a composite hydrogel with multiple synergistic effects in the treatment of psoriasis. The gel contains good biocompatible zinc mesoporous nanospheres, and Ag nanoparticles are evenly dispersed in the nanospheres (denoted as ZnO/Ag or ZA). Silver nanoparticles enhance the immunomodulatory properties of ZA by inhibiting cytokines associated with innate immunity and modulating the STAT3-cyclin D1 pathway through the removal of ROS, thereby suppressing the self-amplifying behavior of cytokines that are linked to adaptive immunity (Figure 2A). Simultaneously, the microspheres are integrated with methotrexate to enable the sustained release of MTX. The MTX–zinc (MTX-ZA) microspheres, modified with nanomicelles and incorporated into Carbopol, form the MTX–zinc hydrogel. The MTX–zinc hydrogel showed a long skin retention time over 24 h (Figure 2B). In the IMQ-induced mouse model, the spleen weight index of the plain MTX-ZA hydrogel-treated group is 8.97 ± 0.53; the value of the MTX-ZA hydrogel-treated group is 2.95 ± 0.13, which showed a better therapeutic effect [50]. Ionic liquids consist of organic cations incorporated with organic or inorganic anions. Recently, ILs formed from choline cations and organic acid anions have gained attention due to their numerous advantageous properties. Choline and pelargonic acid (CAGE) are selected to form ionic liquids. Microemulsion is a thermodynamically stable solution composed of amphiphilic components that contain micrometer-sized droplets. Ionic liquid microemulsion combines both ILs and microemulsion solutions to solubilize poor-solubility drugs and enhance penetration.

Shu et al. developed a thermally responsive hydrogel utilizing an ionic liquid microemulsion for the topical administration of MTX. Poly(N-isopropylacrylamide) (PNIPAM) hydrogels are commonly employed in temperature-sensitive drug delivery systems because they present low critical solution temperatures. Incorporating fibroin protein into the hydrogel structure improves its mechanical strength and adhesive capabilities. The permeability of the MTX/IL-ME hydrogel surged to a plateau of 32% at 16 h compared with 5.1% for the MTX/PBS hydrogel, indicating that the drug permeability of IL-ME increased by six times. Additionally, the MTX/IL-ME hydrogel demonstrated significant antibacterial effects, reducing intracellular Staphylococcus aureus colony counts by 95.6%. In cell viability experiments, cells co-incubated with the MTX/IL-ME hydrogel showed survival rates of 88% at 24 h and 81% at 48 h, indicating that the ionic liquid-based microemulsion hydrogel exhibits characteristics of enhanced permeability, antibacterial activity, and good biocompatibility [52]. Asad et al. prepared Eudragit E100 nanoparticles that contain amino-cationic groups to enhance cell uptake and protect the nanoparticles, thus avoiding the proton sponge effect to deliver MTX. The nanoparticles were embedded into chitosan-based hydrogels. In vitro permeability tests revealed that the concentration of the drug preserved in the skin was limited to 19.95 ± 1.04 µg/cm^2^, with the epidermis retaining 81.33% of the drug over a 24 h period [53].

Pandey et al. used a thin film hydration method to prepare the niosomes in order to improve the solubility of cyclosporine (CyA) and add Carbopol 940 to form a gel. A drug retention study revealed that the deposition of cyclosporine in the stratum corneum and the viable layers was 50.57%, compared with 10.13% of cyclosporine suspension in 24 h. In terms of histopathology, the PASI scores of mice treated with cyclosporine-loading niosome gel decreased significantly [54]. They also prepared a microemulsion-based cyclosporine gel consisting of an oil phase (isopropyl), surfactant (Tween 80), and co-surfactant (isopropyl alcohol). After selecting the best surfactant mixture ratio, microemulsion was added to carbomer gel for local application. Compared with a suspension, the cumulative drug release percentage was improved from 4.2 ± 0.31% to 14.71 ± 0.31%. The amount of cyclosporine retained in the epidermis and viable layers also increased from 12.36 ± 2.2% to 42.36 ± 6.5%, leading to the sustainable treatment of psoriasis [55].

#### 2.2.2. Microneedles

MNs are capable of different types of drug delivery, both small molecules and biological agents, for various kinds of diseases, such as cardiovascular disease, tissue regeneration, and immune-mediated diseases [56]. Invasive and noninvasive soluble microneedles have become the most studied form of microneedles due to their advantages of simple manufacture, safe handling, diverse materials, good biocompatibility, and high drug loading. Once applied to the skin, the needle dissolves to mediate immediate, controlled, or continuous delivery of the drug [57]. Currently, Tekko et al. reported the combination of methotrexate sodium (MTX Na) nanocrystals with soluble microneedles. MTX Na cannot be successfully administered subdermally because of hydrophilicity. The clearance rate of MTX Na is high, requiring more frequent administration to maintain the dose intensity, which may affect patient compliance. The MTX Na microneedles demonstrated excellent mechanical strength and effective penetration in skin simulations, fully dissolving within 20 min after application to porcine skin and delivering approximately one-quarter of the MTX to the psoriasis skin. Deposition of much larger amounts of MTX Na than oral MTX was achieved while minimizing systemic exposure to MTX [58]. Du et al. prepared silica nanoparticles with hollow structures, coated MTX in the cavity, and then adsorbed positive-potential chitosan onto the surface of microneedles. The silica MNs initially release 18% of drugs within 2 h, and the slow drug release process can last for 7 days, thus avoiding an outburst initial release profile and systematic blood vessel circulation [59]. Oxidative stress, NF-κB, and adenosine are linked to signaling pathways involved in psoriatic inflammation. Zhou et al. developed a CD44-targeted MTX prodrug with ROS responsiveness designed to specifically target overactive keratinocytes. The nanocomposite of MTX prodrug combined with two polymers was created using a nanoprecipitation technique. To address the thickened stratum corneum in psoriatic skin, the nanocomposite was incorporated with soluble microneedles by using a micro-molding method. The confocal laser scanning microscope (CLSM) image of HaCaT proved that nanoassemblies can be internalized by keratinocytes through a CD44-mediated pathway. The Franz diffusion cell experiment proved that the MTX prodrug encapsulated in microneedles can release 91.1% within 120 min, indicating that the prodrug can effectively penetrate the epidermis, subsequently inhibiting the hyperproliferation of keratinocytes by blocking the NF-κB pathway. The immunoblot analysis showed a 2.33-fold decrease in NF-kB levels of inflamed skin tissue [60]. Zhao et al. prepared a microneedle array patch with expandable tips (TSMAP), incorporating microneedle tips made of photo-crosslinked hyaluronic acid (MeHA) and a base constructed from biocompatible resin. Using vacuum drying, MeHA is concentrated at the tip of the needle to form an expandable tip that can be effectively loaded onto the tip after being incubated with MTX solution and then dried. Due to its good mechanical strength in piercing psoriasis-like skin, the tip of the loaded MTX can be separated from the matrix and released continuously and slowly as a drug reservoir. The comparative release experiment with fluorescein revealed that the dermal fluorescence signal of TSMAP persisted for 96 h before dissipating, which is significantly longer than that of the subcutaneous injection group [61].

Hadler et al. prepared cyclosporine-coated polyvinyl alcohol (PVA) microneedles using micro-molding and solvent-casting methods. The physical characteristics of the microneedles were investigated, and the drug encapsulation efficiency, release profile, skin penetration, and anti-psoriasis pharmacological activity of microneedles were evaluated. In animal models, the microneedles had good transdermal strength of 630 μm, and within 30 min of insertion into the skin, a more than 60% needle length reduction was revealed, 87% of the drug was released within 60 min, PASI scores and proinflammatory cytokine levels were significantly reduced, and spleen size was significantly reduced. The properties of the microneedles remained stable for 6 months under accelerated conditions [62].

In summary, numerous attempts have been made to deliver the immunosuppressants methotrexate and cyclosporine with microneedles for localized treatment of psoriasis, and experiments have already been conducted on humans. Many microneedles are designed for long-term administration. Over extended periods of administration, inappropriate needle lengths may damage the skin, and the minor injuries caused by microneedles could potentially exacerbate redness and swelling in the affected skin areas, along with the risk of disrupting the epidermal microbial homeostasis. Therefore, when considering patient use, it is advisable to assess the safety of the formulation, skin irritation, and potential toxicity.

#### 2.2.3. Niosomes and Nanoparticles

Researchers have developed a series of nanocarriers to enhance drug solubility and promote penetration, such as liposomes, transferosomes, ethosomes, etc. Yang et al. first proposed ceramide niosomes loaded with methotrexate and niacinamide (MTX/NIC). Niacinamide is a hydrophilic substance that can form hydrogen bonds with MTX and significantly solubilizes MTX. In vivo penetration experiments have shown that ceramides can significantly promote skin penetration and retention. The total skin retention of MTX from MTX/NIC ceramide niosomes was measured at 0.47 ± 0.04 μg/cm^2^, which is 3.36 times greater than that achieved from the MTX suspension. Proinflammatory cytokine levels were markedly reduced in both the LPS-stimulated HaCaT cell model and the imiquimod-induced psoriasis mouse model [63]. Gold nanoparticles (AuNPs) are an excellent option for localized drug delivery systems owing to their editable properties. Bessar et al. prepared functional gold nanoparticles to deliver MTX. The encapsulation efficiency of MTX on the nanoparticle is 70–80%, and the release rate reached 80% within 1 h and 95% within 24 h. Preliminary toxicity tests on keratinocytes showed that AuNPs were non-toxic. The distribution of the conjugate on bare mouse skin was monitored in vivo using UV-Vis spectroscopy following a 24 h administration period. The results demonstrated enhanced MTX penetration into both epidermal and dermal layers when delivered through the gold nanoparticle treatment, as opposed to the application of free MTX. Pentoxifylline has also been reported to have the activity of downregulating psoriasis-related inflammatory markers and can reduce the dose-dependent side effects of cyclosporine [64]. Bhardwaj et al. designed a niosome that co-delivered cyclosporin and pentoxifylline, with a −37.5 mV Zeta potential. Based on the permeation study data, niosomes raised the permeation rate of MTX from 23.4 µg/cm^2^/h to 37.24 µg/cm^2^/h, compared with dispersion. The restricted movement of negatively charged vesicles in the bloodstream is likely due to decreased electrostatic repulsion within the intercellular spaces [65].

#### 2.2.4. Micelles

Micelles are drug delivery systems created through the spontaneous aggregation of amphiphilic surfactant molecules in a solution. With the development of technology, scientists have invented reverse micelles on the basis of micelles, making it possible to load hydrophilic and lipophilic drugs with micelles [66]. MTX can be dissolved in an ionic solution at a physiological pH 7.4 due to its polar molecule essence. An amphiphilic material called DMSAP was synthesized by Zhao et al. The micelles were prepared by adding soybean phosphatidylcholine (SPC) to the DMSAP. MTX was attached to the hydrophilic shell of the micelles using electrostatic adsorption. The micelles were characterized by a diameter of around 100 nm, a positive Zeta potential of +36.26 mV, and an EE of 91.50 ± 1.02%. In 56 h, 44.81 ± 11.22% of MTX infiltrated into the skin in Franz diffusion cells [67].

Riboflavin, a biocompatible aromatic compound, can be integrated into grafted molecules and form hydrogen bonds with methotrexate due to its isoalloxazine ring. Additionally, the positively charged groups on arginine and lysine side chains enable electrostatic interactions with MTX, further enhancing binding. A study was conducted in which a reverse micelle was prepared by isolating MTX in the core of the nanocarrier through an optimized micelle formation method. The dialysis method was used to evaluate the capacity of controlled release. When the content of lysine is increased, the release of the drug can be extended for 48 h, reaching 70% at the endpoint. The release profile of micelles showed no obvious initial burst release, while the free MTX diffused 90% in 2 h [68].

#### 2.2.5. Nanoemulsion

Nanoemulsions, typically with droplet sizes below 500 nm, serve as one of the primary carriers for drug bioavailability progress. The oral administration of cyclosporine often induces systemic toxicity, demonstrating dose-dependent toxic effects. Musa et al. created a cyclosporine nanoemulsion. The system exhibited an IC50 value of 1000 mg/mL in HaCaT cells, indicating no cytotoxic effects. Sterility potential testing yielded negative results for both G+ and G- bacteria. In clinical trials involving healthy volunteers, the formulation demonstrated significant enhancement in skin hydration, with two different formulations increasing water retention by 54.67% and 68.87%, respectively, suggesting excellent moisturizing properties [69].

#### 2.2.6. Ionic Liquids

Ionic liquids (ILs) are “liquid salts” created by combining acids and bases, serving roles such as solubilizers, antimicrobial agents, and permeability enhancers in various biomedical applications. When one component of the ionic pair is an active pharmaceutical ingredient, the resulting ILs are referred to as API-ILs. Previously, several choline-based ILs have been observed to enhance the skin delivery of a range of biological macromolecules, such as choline malate, choline-producing acid choline, and several choline fatty acids. As a new osmotic accelerator, ILs have the advantages of biocompatibility, degradability, customization, stability, and safety [70]. Li et al. use ILs as carrier systems and osmotic accelerators to promote CyA solubility. The potential of choline ricinoleate ([Ch] [Ra]), choline sorbate ([Ch] [So]), choline geranate ([Ch] [Ge]), and choline citrate ([Ch] [Ci]) in the cutaneous delivery of cyclosporine was evaluated. In three of the four ILs, the solubility of CyA increased to 95.5 mg/mL, while its solubility was only 16.4 mg/mL in [Ch] [Ci]. The content of water in ILs significantly influences the permeation efficacy; the maximum efficiency is achieved at a 30% water content. In pharmacodynamics studies, the ILs presented around a 70% reduction in TNF-α and IL-22. Moreover, [Ch] [So] demonstrated no significant cytotoxicity and irritation compared with azone following 7 days of administration [71]. To solubilize and facilitate the infiltrating properties of cyclosporine, Datta et al. prepared a cyclosporine-loaded system by combining choline/geranic acid ionic liquid with Pluronic F127. Compared with free cyclosporine, cyclosporine ionic liquids and ionic liquid gel improved the penetration of cyclosporin in porcine skin models. In rat pharmacokinetic models, the Cmax and AUC values of gel were increased 2.6 and 1.9 times, respectively. In the imiquimod model, the psoriasis lesion area and PASI score were significantly reduced, and changes in skin thickness, blood flow, and percutaneous water loss were reversed [72].

### 2.3. Retinoids

#### 2.3.1. Liposome and Transethosome (Vesicles)

At the cellular level, retinoic acid inhibits the generation of proinflammatory Th17 cells mediated by IL-6, promotes the differentiation of Treg cells, and suppresses excessive immune responses. The limitations of the clinical application of retinoic acid are mainly reflected in low water solubility, photosensitivity, and skin irritation. Intravenous administration increases catabolism, reduces therapeutic effectiveness, and increases systemic side effects [73]. Wang et al. developed a nano-transdermal delivery system comprising all-trans retinoic acid (TRA) and gel. The TRA nanoparticles, synthesized using the reverse micelle method, exhibit strong mitochondrial targeting and valence transition capabilities linked to reactive oxygen species (ROS) scavenging. The TRA gel system, prepared using the thin film dispersion method, can effectively encapsulate the drug. The TRA gel formulation extended drug release to 72 h, whereas the free TRA gel released nearly all of the drug within 8 h. Additionally, skin retention improved by 1.81 times. The EGF induction and the H_2_O_2_ induction showed that the TRA gel efficiently downregulated inflammatory levels of HaCaT cells and alleviated oxidative stress. The results of the IMQ-induced in vivo model showed that the TRA gel significantly alleviated psoriasis symptoms [74]. Retinoic acid systems often lead to significant systemic side effects, and their topical application is restricted due to low water solubility and skin irritation. To address these issues, Zhao et al. developed a fatty acid vesicle encapsulating retinoic acid (Tre-FAV). The drug encapsulation reached 84.26 ± 0.816%, and the surface charge was −28.9 ±1.92 mV. The skin retention of Tre-FAV increased by 2.89 times compared with the solution form. In mouse models, the topical application of Tre-FAV demonstrated superior anti-psoriatic effects, effectively reducing papules, erythema, and epidermal thickness. Spleen weight decreased post-treatment, and serum levels of proinflammatory cytokines were significantly reduced [75]. Bexarotene is one of the retinoids that exerts a better therapeutic effect on psoriasis, but its poor solubility and skin irritation make it difficult to administer via the topical route. In order to overcome these shortages, Saka et al. incorporated it into liposomes to solve these problems. This study focused on optimizing the preparation of bexarotene liposomes by adjusting key factors, including the compound ratio, sonication time, drug content, and loading capacity. The optimized liposomes, with a particle size of 67.8 ± 7.15 nm, were integrated into a gel, achieving an encapsulation efficiency exceeding 95%. Pharmacology studies using the BALB/c mouse psoriasis plaque model induced using imiquimod confirmed that bexarotene liposomes can effectively treat psoriasis by reducing scale and inflammation without any toxicity. After treatment, TNF-α, IL-17, IL-22, IL-23, and IL-2 decreased by 1.94, 1.96, 2.49, 1.40, and 2.14 times compared with a negative control [76]. At present, the mainly oral formulations of acitretin A in the market for psoriasis are not effective and have serious teratogenicity and other systemic side effects. Skin irritation is one of acitretin’s adverse reactions. Kaur et al. developed gel formulations based on elastic liposomes and ethosomes, which have deformable capabilities and higher skin permeability compared with nanoparticles. Elastic liposomes and ethosomes were integrated with Carbopol 934 to assess the appearance. The skin irritation score of liposome gel (lipogel) and ethosome gel (ethogel) was below 0.9, while the commercial acitretin gel (plain gel) caused erythema and edema. An in vivo localization test showed that the skin localization index values of lipogel and ethogel were higher at 39.5% and 36.1%, compared with plain gel, which was 17.5%. Mechanistic studies using SEM analysis also confirmed the potential for better skin localization. The in vivo experiment revealed that the novel carriers had a better therapeutic effect. Compared with the negative control (13.6 ± 4.2%), the groups treated with elastic lipid gel and ethanol gel showed significant differentiation in the epidermis, as indicated by the orthokeratosis percentages (65.1 ± 5.6% and 58.4 ± 3.2%, respectively) [77]. Bevinakoppamath et al. developed acitretin-loaded transethosomes incorporated into xanthan gel to address the challenges of acitretin’s low water solubility and poor skin penetration. The application of a Box–Behnken design directed the optimizing process of the formulation of transethosomes, which were prepared using the cold method with varying concentrations of lipids, ethanol, and surfactant. Ex vivo permeation experiments showed that transethosome delivered 914.51 ± 16.53 μg/cm^2^ of the drug compared with the 700.31 ± 0.45 μg/cm^2^ delivered by conventional gel. The minimum inhibitory concentration (MIC) was 12.5 μg/mL, and the minimum bactericidal concentration (MBC) was 50 μg/mL, indicating that transethosome gel has optimized antibacterial activity [78].

#### 2.3.2. Nanoparticles

Kshirsagar et al. prepared tazarotene-loaded PLGA nanoparticles using nanoprecipitation that targets skin and hair follicles. FT-IR characterization of the nanoparticles showed that there was no interaction between tazarotene and PLGA. The dialysis method was used to evaluate the in vitro release of nanoparticles, which can release 69.38 ± 2.26% in 36 h fitting in the Higuchi model. IVPT experiments based on porcine skin showed that the active form of tazarotene, tazarotene acid, was detected in the skin and that tazarotene nanoparticles were more effective at maintaining bioconversion capabilities and targeting hair follicle delivery in comparison with the solution form. On account of the minimal particle size of PLGA nanoparticles, the follicular delivery was 1.37 ± 0.16 µg/cm^2^, demonstrating overall higher penetration efficacy compared with the solution form [79].

#### 2.3.3. Solid Lipid Nanoparticles

Solid lipid nanoparticles (SLNs) and nanostructured lipid carriers (NLCs) are arousing scientists’ interest in solving the problems of BCS (Biopharmaceutical Classification System) Class II and BCS Class IV drug load and release. Lipids with different properties can play a role in increasing solubility and improving biocompatibility and biodegradability [80]. When the lipid nanoparticles are applied to the skin surface, the water in the lipid nanoparticles evaporates, and the lipid particles form a bonding layer that blocks the skin surface. Enhancing the hydration of the stratum corneum can loosen its compact structure, thereby facilitating the entry of the drug into deeper layers and underlying blood vessels [81].

Aland et al. prepared tazarotene SLNs using thermal homogenization. The effects of eight parameters (surfactant type and concentration, drug/fat ratio, ultrasonic time, etc.) on the particle size and encapsulation rate were investigated using Taguchi’s orthogonal array. After determining the types of excipients, the optimal concentration was determined under the central composite design. The in vitro release experiment exhibited that the release of tazarotene SLNs in 60 min was 98.12 ± 1.52%, which was better than 42.12% of a single drug; this result was consistent with the zero-order kinetic process. The release fluxes of tazarotene SLNs loaded in Carbopol 934p gel and tazarotene gel on the membrane and skin were compared. The measurement of the flux value of the SLN gel was higher than that of the commercially available gel, indicating that it had better skin barrier penetration performance. The viscosity of the SLN gel preparation is 5.98 × 10^3^ ± 0.34 × 10^3^ cp. It has good skin adhesion and can provide good contact with the skin affected by the lesion; moreover, the preparation is stable for 3 months [82].

### 2.4. Vitamin D Analogs

#### 2.4.1. Hydrogel

Vitamin D and its analogs participate in regulating cell differentiation and proliferation. They also modulate apoptosis and exhibit immunomodulatory properties. Over the years, the potential of vitamin D analogs for topical psoriasis treatment has been developed well [83]. Recent advancements in nanocarrier-based delivery systems have enhanced the bioavailability of these analogs [84]. Pradhan et al. created and optimized a calcipotriol-loaded nanostructured lipid carrier (CPT-loaded NLC) through the Box–Behnken design. The NLCs featured a 123.60 ± 1.21 nm average particle size, 85.31 ± 1.18% encapsulation efficiency, and −36.8 ± 8.85 mV Zeta potential and spherical structure. The release profile indicated that the sustained release aligned well with the Higuchi model. Additionally, the penetration experiments proved that the amount of drug entering the systemic circulation was minimal and considered negligible. Compared with pure CPT gel, the retention of CPT delivered by NLCs in the stratum corneum and viable layers was enhanced by 1.57- and 3.67-fold [85].

Nanofibers (NFs) can enhance skin hydration and increase drug bioavailability. Mimicking the extracellular matrix, nanofibers possess regenerative properties, support cell adhesion, and hold drug release in a controlled way at psoriatic sites compared with conventional treatments. Researchers have developed and tested nanofibers loaded with tazarotene (TZT) and calcipotriol (CPT) combined with carbohydrate-based hydrogel membranes. Nanofibers were prepared from a polyvinyl alcohol/polyvinylpyrrolidone (PVA/PVP) K-90 blend polymer using the electrospinning method, and then the nanofibers were mixed into the Carbopol group to form a hydrogel film. These formulations were fully biodegradable within 2 weeks of application, demonstrating good biocompatibility. After 72 h, the drug release rate of TZT-CPT-NFs was 95.68 ± 0.03%, fitting the Higuchi model. TZT-CPT-NF gel membranes showed anti-psoriasis activity in psoriasis Wistar rats, decreasing the relative epidermal thickness percentage to 58.23% [86].

#### 2.4.2. Microneedles

Dai et al. prepared calcipotriol monohydrate (CPM) into nanosuspensions (NSs), which can increase the dissolution rate and saturation solubility of drugs. These CPM NSs were incorporated into a three-layer dissolving microneedle patch (MAP) composed of poly(vinylpyrrolidone) and poly(vinylglycol). The MAP was pre-treated using a 3D-printed substrate layer with rapidly dissolving tips that have excellent mechanical strength and insertion capability (Figure 2C). The needle height reduction in the CPM MAPs was 9.29 ± 2.87%, indicating that only the drug-loaded tips penetrated the epidermis. The tips of the MAP fully dissolved within 15 min (Figure 2D). The efficacy of this innovative MAP was evaluated in SD rats with imiquimod-induced psoriasis. The skin thickness in the CPM-NS-MAP group measured 1.35 mm, outperforming the results achieved with commercial ointments [51].

#### 2.4.3. Micelles

In the psoriatic skin microenvironment, there is a high concentration of reactive oxygen species (ROS). Nanocarriers based on ROS can facilitate the targeted release of drugs at the lesion site [87]. Hua et al. developed an amphiphilic ROS-sensitive material (mPEG-ss) to encapsulate calcipotriol (CPT), forming ROS-responsive micelles (PSCs). Under pathophysiological conditions, the concentration of hydrogen peroxide ranges from 50 to 100 μM, and the hydrogen peroxide response concentration of PSCs falls within this range, indicating that these nanomicelles can release drugs under pathological conditions. Compared with the positive control, skin slices demonstrated that the PSC group significantly reduced immune cell infiltration. Additionally, it markedly lowered the levels of IL-6 and TNF-alpha [88].

#### 2.4.4. Nanoemulsion

Many exopolysaccharides (EPSs) have an emulsifying ability and the potential to become Pickering emulsions. Wang et al. identified the EPS of a strain of Marine bacteria FYS and affirmed the best emulsifying conditions (pH and temperature). They then prepared a novel EPS/CT (calcipotriol) Pickering nanoemulsion (ECN) to improve the poor skin permeability of calcipotriol. The findings revealed that ECN is an O/W-type emulsion with a dynamic viscosity of around 101.8 ± 8.5 mPa·s. The droplet size and Zeta potential were measured at approximately 170 nm and −30 mV. Under conditions of pH = 5–6 and 25 °C, the particle size remained within the range of 170.8–287.0 nm without phase separation over 2 months. The EE of ECN was approximately 90%, and the cumulative drug release achieved 95.0% over 36 h. Histological analysis using H&E staining demonstrated that the therapeutic efficacy of ECN was on par with that of Daivonex (CT scalp solution) [89].

## 3. Biomacromolecules

### 3.1. Protein and Peptide

#### 3.1.1. Protein and Peptide Delivery

The potential of proteins for treating psoriasis has been explored as a promising strategy in recent years. Previously, the C-terminal peptide of chemokine-like factor 1 (CKLF1) has demonstrated protective effects against psoriasis by inhibiting the MAPK pathway and reducing inflammation [90]. Similarly, a selenium-enriched yeast peptide fraction (SeP) has demonstrated anti-inflammatory effects in psoriasis treatment by inhibiting both the MAPK and NF-κB pathways, although the specific molecular targets of these peptides have yet to be fully elucidated [91]. Moreover, the signal transducer and activator of transcription 3 (STAT3) participated deeply in regulating the pathogenesis of psoriasis and can be targeted to inhibit its development. Asai et al. reported a water-soluble STAT3-inhibiting peptide, caveolin-1 (CAV-1), which subsequently suppresses cytokines, such as IL-22, and inhibits angiogenesis [92]. Another peptide identified for alleviating psoriasis symptoms is Sprouty1 (SPRY1). SPRY1 functions as an inhibitor of receptor tyrosine kinases (RTKs), thereby attenuating various proinflammatory signaling pathways, including MAPK and STAT3 [93]. A nanoparticle-based peptide APTstat3-9R that inhibits STAT3 was recently reported [94]. Additional preparations have also been reported. Javia et al. developed liposome-encapsulated antimicrobial peptides (AMPs) that demonstrate antibacterial and anti-inflammatory properties [95]. Similarly, Zhao et al. designed a dual-functional microneedle incorporating M-CSF and IL-13, which exhibited potential for immunotherapy and multi-drug release to modulate macrophage phenotypes [96]. In summary, most current strategies utilizing proteins and peptides focus on inhibiting the inflammatory response.

#### 3.1.2. Gene Editing

Protein-based therapeutics have gained significant traction in the pharmaceutical sector since the FDA’s initial approval of recombinant human insulin for diabetes management in 1982. The range of protein drugs, mainly monoclonal antibodies and cytokines, also including enzymes and peptides, has expanded rapidly. However, the inability of proteins to penetrate cell membranes limits their effectiveness to extracellular targets, posing a challenge for intracellular applications. A major focus in therapeutic development is creating protein drugs with high target efficiency. CRISPR-Cas9, a transformative genome-editing technology, has brought to light the critical demand for effective delivery systems capable of transporting Cas9 ribonucleoprotein complexes into the nuclei of target cells. In the last ten years, researchers have developed a range of delivery strategies, encompassing both viral and non-viral methods, to facilitate Cas9 RNP-mediated gene editing. Despite the high efficiency of viral vectors, they are associated with significant risks, including immune system activation, unintended genomic integration, and the potential to induce oncogene expression. In contrast, non-viral systems, including lipids, inorganic nanoparticles, and polymers, are safer and have gained widespread use in Cas9 RNP genome editing [97].

Wan et al. developed functional microneedle arrays to co-deliver dexamethasone PLGA nanoparticles and NLRP3 specific-Cas9 ribonucleoprotein (RNP) polymer nanocomplexes in 2021, targeting the NLRP3 gene to disrupt NLR inflammasomes. Disrupting inflammasomes can alleviate the common off-target problem appearing in corticosteroid treatment. On the other hand, Dex promoted the expansion of nuclear poles, which provided convenient entry of gene editing agents. The presence of Dex increased the indel frequency from 29.6% to 36.2% in DC2.4 cells and 19.1% to 31.7% in 3T3 cells. When microneedle patches were administered to mice, the results showed that the indel frequency was 17.2%, and the skin disorders were vastly relieved [98]. Three years later, the team collaborated with Tan et al. to identify a highly efficient dendrimer delivery vehicle constructed with phenyl-boronate and lipoic acid, also targeting NLRP3 for psoriasis. They screened a series of ratios of phenyl-boronate and lipoic acid to achieve the highest intracellular delivery efficiency. Pharmacodynamics results showed that 33.7% of NLRP3 locus had been disrupted, and psoriasis symptoms had obviously improved equally to calcipotriol ointment [99].

### 3.2. Nucleic Acid

#### 3.2.1. RNAi Therapy

Targeting the RNA interference (RNAi) pathway offers a way to modify disease-related biological processes in various medical conditions, including autoimmune disorders. Large-scale genome-wide association studies have identified over 80 genetic loci linked to psoriasis susceptibility [100]. In psoriasis, RNAi can suppress the expression of molecules involved in the disease’s pathogenic mechanisms. However, delivering siRNA to the target site without adverse effects remains challenging. Barriers to RNA delivery include carrier collapse in the bloodstream, renal clearance, binding to plasma proteins, entrapment by the mononuclear phagocytic system (MPS), and membrane impermeability. Intracellularly, endosomal entrapment after cellular uptake is a major obstacle [93]. A successful siRNA delivery system should fulfill several essential requirements: (1) protect siRNA against degradation within the body; (2) traverse the endothelium to access target tissues; (3) allow for regulated siRNA release; (4) promote efficient cellular uptake; and (5) ensure proper endosomal escape and cytoplasmic delivery of siRNA to enable the formation of the RNA-induced silencing complex (RISC) for functional activity. Progress in biomaterials science has driven the creation of synthetic lipids and polymers that effectively tackle numerous obstacles in siRNA delivery. These materials provide a foundation for designing safe, effective, and clinically viable siRNA delivery systems. Significant progress has been made in RNAi-based therapies for psoriasis [101].

Tetrahedral framework nucleic acid (tFNA) has become an excellent transdermal carrier for gene transport due to its noninvasive and editable properties. Tannic acid (TA) is a natural compound that has anti-inflammatory and antioxidant activity. TA can incorporate with nucleic acids through hydrogen bonds and mediate the size regulation of nucleic acid delivery nanomaterials. Zhang et al. created a complex assembled using TA, tFNA, and siRNA named STT. STT enters the lysosome through caveolin protein-mediated endocytosis and releases the drug via hydrogen bond dissociation under the regulation of lysosomal acid. STT inhibited the secretion of interleukin-17 and 23 and TNF-α by silencing the NF-kB pathway, breaking the closed-loop inflammatory cycle. Additionally, STT helps restore the skin’s immune balance by curbing the overgrowth of keratinocytes, decreasing the secretion of numerous inflammatory mediators via dendritic cells, and limiting the transformation and differentiation of mDCs [102].

Xin et al. designed self-assembled “sandwich” structured nanoparticles based on methoxy-pegylated poly-histidine (mPEG-pHis) and methoxy-pegylated polylactic acid (mPEG-PLA) that have the ability to adsorb specific disease proteins, allowing the nanoparticles to accumulate in peripheral inflammatory immune cells. After intravenous injection, psoriasis-specific proteins and autoantigens in peripheral blood automatically cover the nanoparticles, enabling the uptake percentage of CD11b+ to increase by 50%. The protein corona allowed the nanoparticles to bind to formylpeptide receptors and Toll-like receptors (TLR-4), facilitating therapeutic localization in PBMCs and ultimately facilitating the localization of the nanoparticles into psoriasis lesions, supplied by the results of flow cytometry. Systemic oxidative stress triggers the step release of “sandwich” nanoparticles. MTX and siRNA-TNF-α combine to play anti-inflammatory effects, causing a reduction in the percentage of proliferating HaCaT cells from 19.1% to 10.1%. The catabolism of histidine can also promote the sensitivity of cells to MTX by upregulating folate 2.5 times. Considering all of this, nanoparticles can block the skin-homing effect of immune cells and regulate the systemic immune microenvironment [103]. Lee et al. developed a PLGA nanoparticle that delivers siRNA. Researchers utilized bioengineering technology (heat laser) to promote skin absorption. To improve endosomal escape and minimize nanocarrier leakage, the nanoparticles incorporated a cationic surfactant, facilitating binding with anionic siRNA. The knockdown efficiency of interleukin-6 levels in keratinocytes and macrophages was around 60% and 70%. In the Franz experiment, the fractional laser promoted the skin deposition of nanoparticle siRNA up to 3.3 times and reduced IL-6 by 56%. The laser-assisted nanocarrier alleviates erythema and squamous lesions in psoriatic dermatitis and reduces epidermal hyperplasia and immune cell infiltration [104].

**Figure 3:**
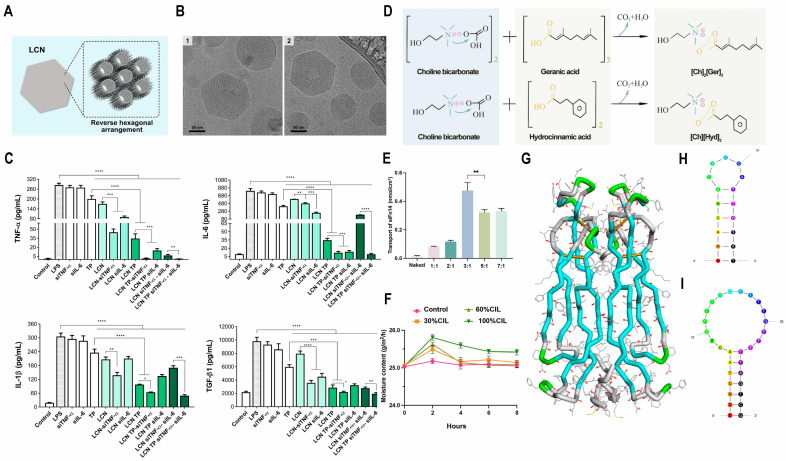
(**A**) Schematic structure of LCNs. (**B**) Cryo-Transmission Electron Microscopy micrograph of LCNs prepared (1) without PAH polymer and (2) with PAH polymer (scale bar: 50 nm). (**C**) The quantitative results of downregulation efficiency of proinflammatory cytokines (TNF-α, IL-6, IL-1β, and TGF-β1). Data shown are means ± SD (n = 3/3 independent tests); two way-ANOVA, Tukey’s post-test: * *p* < 0.05; ** *p* < 0.01; *** *p* < 0.001, and **** *p* < 0.0001. Reproduced from Ref. [106]. (**D**) Schematic representation of formulation preparation of complex ionic liquids. (**E**) Quantification of the fluorescence intensities of different CIL preparations, implying the ability of keratinocyte internalization. Different columns in colors represent different ratios of complex siFn14-ionic liquid preparations. Data are expressed as mean ± SD. ** *p* < 0.01. (**F**) Rate of transdermal water loss of siFn14-IL in 8 h. Reproduced with permission from Ref. [107]. (**G**) Three-dimensional structure of human IL-17A homodimer. The different colors represent different secondary structures of protein (blue represents β-pleated sheet; green represents β-turn). The schematic representation of the structure of (**H**) M2 aptamers and (**I**) M7. Multiple colors were used to distinguish bases. Reproduced with permission from Ref. [108].

Liquid crystal nanoparticles (LCNs) present a compelling option for topical drug delivery owing to their well-organized internal structure, expansive surface area, and resemblance to the skin’s architecture. Silvestrini et al. used poly(allylamine hydrochloride) (PAH)-modified LCNs with reverse hexagonal microstructure (Figure 3A,B) to entrap triptolide (TP), TNF-α, and IL-6 siRNA on its surface, aiming at local co-delivery in psoriasis treatment. When LCNs were embedded in hydroxyethyl cellulose hydrogels (LCN-TP), their co-delivery capacity was higher. The results of the IVPT experiment showed that TP was detected in pig skin, and the dermal distribution increased more than 20 times. In the efficacy study, LCNs showed good compatibility and rapid internalization, reducing the expression levels of TNF-α by 11.4-fold, IL-6 by 3.88-fold, and IL-1β by 2.63-fold in lipopolysaccharide (LPS)-stimulated macrophages (Figure 3C) [105]. Viegas designed NLCs for the delivery of tacrolimus (TAC) and siRNA-TNF-α together, with a particle size of approximately 230 nm and a surface charge of +10 mV. In vitro skin penetration and retention experiments revealed that NLCs efficiently transport siRNA through the stratum corneum and improve the retention of tacrolimus within this layer. Finally, the expression of TNF-α was shown to be decreased by about seven times in the psoriasis mouse model. Therefore, TAC and TNF-α siRNA together can exert an amplifying effect on psoriasis [106].

Suzuki et al. prepared hybrid nanoparticles composed of lipids and polymers, which can effectively entrap the photosensitizing agent meso-tetraphenyl porphyrin disulfonate (TPPS2a) and the siRNA (PLNs). Compared with the TPPS2a solution, PLNs had a 1267-time reduction in skin permeation, while skin retention was similar. This means that PLNs retain most drugs in the psoriasis lesion without allowing them to enter the systemic circulation. Photochemical internalization (PCI) technology can decompose endosome membranes after the photosensitizer (PS) is activated via light and promotes endosomal escape. Following the application of this nanoparticle to an imiquimod-induced mouse model, a 1.38-fold reduction in TNFα expression was observed. When photo-assisted-internalization (PAI) is exerted at a dose of about 75 J/cm^2^, a 4-fold reduction can be observed. These findings suggest that PAI significantly enhances RNA delivery [109].

ILs are capable of promoting permeation and maintaining the integrity of biomacromolecules such as siRNA. The complex ionic liquid (CIL) can exhibit better solubility and stability through the combination of ionic liquids with different characteristics. The elevated expression of fibroblast growth factor-inducible molecule 14 (Fn14) in psoriatic lesions is strongly associated with psoriasis progression. To tackle this, Li et al. created an ionic liquid to enhance the transdermal delivery of siFn14 (Figure 3D) into the skin (Figure 3E,F), aiming to modulate psoriasis-related inflammatory responses. siFn14-IL exhibits excellent skin-hydrating properties, ensuring efficient and prolonged release (Figure 3F). The findings revealed that siFn14-IL markedly decreased Fn14 expression, achieving nearly twice the reduction compared with DMSO-siFn14 and naked-siFn14. Additionally, siFn14-IL demonstrated significantly higher effectiveness, surpassing DMSO-Fn14 and naked Fn14 by 11.43-fold, 10-fold, and 2.25–2.39-fold in lowering EGF, IL-6, and K17 levels, respectively [107]. NFKBIZ, which produces IκBζ, a protein that inhibits NF-κB, has been confirmed to directly regulate the transcription of genes associated with psoriasis. These products are stimulated by inflammatory cytokines such as TNF-α, IL-17A, and IL-36, which play critical roles in inflammatory signaling, neutrophil chemotaxis, and leukocyte activation. Notably, in psoriasis patients, high NFKBIZ occurs simultaneously with abnormal surged expression of interleukins 17 and 36. Targeting NFKBIZ through localized silencing could offer a promising therapeutic strategy, potentially expanding the patient population that benefits from this treatment approach. Mandal et al. evaluated the physicochemical properties and delivery efficiency of various ionic liquid combinations. The mixtures of choline with geranic acid (CAGE) and choline with phenylpropanoic acid (CAPA) demonstrated the highest levels of siRNA accumulation in the epidermis. The knockdown efficiency of IL-siRNA was assessed by measuring the fold change in GAPDH expression. Compared with naked siRNA and untreated mice, the GAPDH expression levels in the IL-siRNA-treated group were reduced by 4.5- and 8.6-fold, respectively. Protein quantification results also showed a two-fold decrease in GAPDH protein expression [110].

#### 3.2.2. miRNA Delivery

MicroRNAs (miRNAs), which are indispensable for RNA silencing and post-transcriptional gene regulation, are short RNAs with no coding function. As a result, the expression of specific miRNAs can affect T cell homeostasis and the abnormal proliferation of tissue cells. Studies have confirmed more than 250 miRNAs with dysregulated expression in psoriasis patients, many of which play roles in controlling key cellular functions. These processes include cell proliferation, differentiation, apoptosis, cytokine production, and the disruption of T cell subpopulation balance. Therefore, targeting miRNAs has emerged as a promising therapeutic approach for psoriasis [111]. Wang et al. discovered that serum exosomes with a high miR-6785-5p content are extensively taken up by abnormal keratinocytes at psoriatic lesion sites. Through transfection experiments, the expression of MNK2 was closely related to miR-6785-5p. Silencing the MNK2 gene effectively suppresses the overexpression of keratinocytes. Investigating the relationship between miRNAs and potential targets in psoriasis could provide more advanced therapeutic strategies for the treatment of psoriasis [112]. The content of miR-125b in psoriatic skin is significantly downregulated. Increasing its expression has been demonstrated to inhibit cell proliferation and boost the levels of multiple differentiation markers. Han et al. selected FNA to deliver miR-125b. FNA-miR-125b enhanced the cellular uptake of miR-125b, which can be observed in mouse skin after 24 h. In a skin disorder mouse model, topical application of FNA-miR-125b reduced the expression of downstream targets STAT3 and TNF-α while also decreasing epidermal thickness and PASI scores [113].

#### 3.2.3. Oligonucleotide Delivery

Oligonucleotides can modulate gene expression through multiple mechanisms, including RNA interference (RNAi), RNase H-mediated RNA degradation, splicing regulation, non-coding suppression, transcription activation, and targeted editing. This molecule has the therapeutic potential to match many indications. Recently, several oligonucleotide-based drugs have been approved for marketing [114]. Iontophoresis (IP) therapy can play a therapeutic role by opening intercellular links mediated by weak electricity to deliver macromolecule drugs. However, psoriasis skin thickening hinders this process. Fukata et al. used IP to facilitate peptide (AT1002) delivery. AT1002 is a six-amino-acid synthetic peptide possessing the ability to open the tight junction. Before the introduction of NF-κB oligodeoxynucleotide into IP, the AT1002 analog was pre-treated, which can penetrate the oligodeoxynucleotide into psoriatic skin. IP can enhance the intradermal permeation by 7.8-fold. Moreover, IP has been found to significantly inhibit the upregulation of psoriasis-induced inflammatory cytokine mRNA and improve epidermal hyperplasia [115]. The study of gene regulatory therapy for skin-related diseases is rarely explored, in part due to inefficiencies in systematic delivery. Fang et al. designed a bottle brush polymer–antisense oligonucleotide (ASO) conjugate, called pacDNA, to target the interleukin 17 receptor A. Following systemic administration, pacDNA was taken up by the skin cells and appeared in both the epidermis and dermis. The pacDNA conjugate also appeared in the immune cells infiltrating the skin. Enhanced skin absorption and preservation facilitated significantly lower oligonucleotide doses to achieve effects comparable to those of conventional formulations. In a mouse model, pacDNA was found to efficiently bind to its target, lowering the level of IL-17, with a maximal reduction of 90–100% under high-dose treatment. Additionally, pacDNA decreased the total PASI score by 60% [116].

#### 3.2.4. DNA Aptamers

Nucleic acid aptamers are short, single-stranded DNA or RNA molecules specifically selected for their ability to bind to particular targets. In comparison with traditional antibodies, they offer advantages such as compact size, structural flexibility, rapid chemical synthesis, diverse chemical modification options, high stability, and low immunogenicity. Aptamers have already been utilized for the targeted delivery of siRNA, miRNA, and conventional drugs [117]. Previous studies have demonstrated that the M2 and M7 anti-IL-17A DNA aptamers are capable of inhibiting IL-17 activity in vitro. Shobeiri et al. investigated the potency of IL-17A aptamers M2 and M7 (Figure 3G–I) in psoriasis treatment. The aptamer-loaded hydrogel was applied to the backs of the mice, causing mRNA levels of inflammatory markers to downregulate, including calcium-binding protein S100A9. Their study found that high concentrations of M2 and low concentrations of M7 notably decreased the PASI score and spleen-to-body weight ratio. Compared with RNA aptamers, ssDNA aptamers exhibit greater stability, making them a promising option for psoriasis treatment [108].

### 3.3. Monoclonal Antibody

#### 3.3.1. Anti-TNF Agents

TNF-α inhibitors, mainly including Etanercept, Infliximab, Adalimumab, Certolizumab, and Golimumab, are the earliest approved monoclonal antibodies for relieving psoriasis symptoms. These biological agents are usually used to modulate TNF-related signaling pathways and cellular responses so that psoriasis can be alleviated. In particular, Etanercept has been demonstrated to be an effective biological agent for moderate and severe psoriasis treatment [118]. Infliximab has been clinically characterized by a prolonged duration of efficacy and a rapid clinical response. Adalimumab therapy affects the innate immune system in the initial stages of treatment, while alterations in the adaptive immune system occur subsequently. Extensive clinical trials and research have demonstrated the high efficacy of these treatments in managing plaque psoriasis, significantly alleviating epidermal inflammation and improving the PASI score [119]. Certolizumab pegol, a pegylated recombinant antibody targeting TNF, is particularly valuable for women of childbearing age due to its minimal side effects in the breastfeeding phase [120]. Golimumab, an IgGκ monoclonal antibody, can be administered subcutaneously or intravenously. It functions by binding to human TNF-α in two ways, preventing TNF-α from interacting with its receptors [121]. However, it is also reported that these mAb drugs can induce adverse reactions such as infection, infusion reactions, and adverse skin effects at the injection site. To avoid adverse reactions concerned with injection, modifying the route of administration of mAb drugs represents a viable therapeutic option. For instance, IP (iontophoresis)-mediated administration successfully delivered Etanercept into psoriatic skin and resulted in the inhibition of inflammatory signals. Furthermore, this method circumvents the inflammatory response that is commonly associated with subcutaneous injections in psoriatic lesions [122].

#### 3.3.2. Anti-IL-17 Agents

IL-17 is an important driver of psoriasis and a highly regarded therapeutic target in psoriasis. By inhibiting the IL-17 pathway, IL-17 blockers are effective in treating psoriasis. To date, the FDA has approved four biologics targeting this pathway: Secukinumab, Ixekizumab, Brodalumab, and Bimekizumab. Clinical trials indicate that Secukinumab and Ixekizumab, both anti-IL-17A agents, are used for plaque psoriasis and psoriatic arthritis. These agents may cause mild side effects such as infections, headaches, and neutropenia [123,124]. The FDA approved the IgG2 monoclonal antibody Brodalumab for severe psoriasis treatment. Brodalumab can bind to interleukin-17 receptors tightly and exerts its function by blocking a series of IL-17 activities differentiated by subtypes, IL-17A, C, E, and F, thereby suppressing the downstream inflammatory effects mediated by IL-17RA. However, Brodalumab can cause nasopharyngitis, which limits its use [125]. Bimekizumab is a dual inhibitor of IL-17A and IL-17F by binding to their mutual amino acid residues. However, using Bimekizumab increases the risk of oral candidiasis compared with other treatments [126]. Considering that the systemic administration of IL-17 mAbs may lead to immune suppression and infection risk, the development of the localized delivery of IL-17 with a strong capacity for lesion penetration is promising to reduce lesion progression and doses. Wu et al. integrated IL-17 mAbs and biocompatible material MXene with a hyaluronic acid microneedle array to create microneedles for psoriasis treatment, which were photo-thermally dissolvable. Demonstrating satisfying mechanical properties and great biocompatibility, these microneedles can easily pierce the skin to deliver mAbs to subcutaneous tissues with minimal immune response. This strategy can inhibit lesion progression, reduce a systemic dose, and may greatly improve drug safety in human bodies [127].

#### 3.3.3. Anti-IL12/IL23 Agents

Ustekinumab is the only drug approved by the FDA for psoriasis that binds to the IL-23 p40 subunit. Some studies have shown that Ustekinumab’s effects can last for a long time (more than three years). Moreover, when treating plaque psoriasis, the treatment effect of Ustekinumab is superior to that of Etanercept (TNF-α inhibitor), which is reflected in higher treatment compliance. Ustekinumab demonstrated a high level of efficacy with just two injections administered over a 12-week treatment period. It is also effective in treating psoriatic nail diseases. Regarding side effects, Ustekinumab treatment may lead to upper respiratory tract infections, nasopharyngitis, neutropenia, and headaches, though it generally causes fewer adverse reactions compared with TNF-α inhibitors [128].

IL-23 inhibitors are mAbs that bind to and inhibit IL-23, thereby reducing the production of psoriasis-related cytokines. The IL-23 mAbs approved by the FDA include Guselkumab, Tildrakizumab, and Risankizumab. Compared with Adalizumab, Guselukumab performed better in scalp, palm, and plantar psoriasis treatment, which helps doctors make treatment decisions for patients with psoriasis in these hard-to-treat areas. Both Tildrakizumab and Risankizumab have good efficacy in moderate to severe psoriasis, especially in decreasing plaque psoriatic lesions, and demonstrate long-lasting efficacy, significantly improving the quality of patients’ lives. Risankizumab binds to the interleukin-23 p19 subunit in a specific manner. The bond inhibits IL-23-related proinflammatory cytokine and chemokine secretion, such as JAK and STAT signaling proteins. Specifically, Risankizumab prevents IL-23-mediated STAT3 phosphorylation. To moderate adverse reactions caused by intravenous or subcutaneous administration, an oral Interleukin-23 Receptor Antagonist Peptide has been developed. This peptide successfully reduced the level of psoriasis, with similar responses observed with several of the approved injectable biologics for psoriasis. However, further study is required to evaluate the sustained security and potency of this treatment [129].

#### 3.3.4. Anti-IL-36 Agents

IL-36 specializes in generalized pustular psoriasis (GPP). Two IL-36 receptor monoclonal antibodies, including Spesolimab, have demonstrated strong potency in treating GPP and palmoplantar pustulosis (PPP) in clinical trials. Spesolimab was approved by the FDA in 2022. In the treatment of GPP, it was able to greatly improve the PASI score. Furthermore, a high dose of Spesolimab exhibited a strong effect on GPP flare prevention. IL-36 mAbs can cause nasopharyngitis and headaches for both single- and multiple-dose administration. Infusion-related reactions were also found in clinical trials of a multiple-dose administration of Spesolimab, which is a common issue related to mAbs. Diverse delivery methods, such as oral delivery and topical delivery, are necessary for mAb drug treatment in the future [130].

## 4. Cell-Based Therapy

### 4.1. Mesenchymal Stem Cell

Mesenchymal stem cell therapy (MSC therapy) is a cell-based approach for managing inflammatory diseases. MSCs possess strong immunosuppressive properties, inhibiting T cell maturation and altering the behavior of immune cells, as evidenced by extensive clinical data. The safety of MSCs remains controversial. In addition, the industrialization of homologous MSCs is high-cost, and there are problems with storage and aging during cell expansion. Therefore, the clinical application of MSCs is challenging, and pulmonary embolism is prone to form after intravenous administration. Compared with MSCs, extracellular vesicles derived from mesenchymal stem cells (MSC-EVs) not only share similar immunomodulatory capabilities but also offer benefits such as low immunogenicity and high plasticity [131].

The extracellular matrix (ECM) undergoes remodeling via matrix metalloproteinase-13 (MMP-13), a process vital for normal functions such as wound healing and angiogenesis. Disruption of this process is associated with the development of various inflammatory diseases. Ren et al. noted a marked rise in MMP-13 expression in skin lesions of an IMQ-induced mouse model and found that TNF-α boosts MMP-13 levels in keratinocytes via the NF-κB signaling pathway. Following the co-culture of hUC-MSCs with THP-1 cells or PMA-induced THP-1 cells in vitro, TNF-α levels in the supernatant dropped, while IL-10 levels rose significantly. IL-10, a crucial immunomodulatory cytokine, plays a pivotal role in suppressing inflammatory immune responses, mainly by inhibiting the activation of immune-related cells. When hUC-MSCs were intravenously administered to IMQ-induced mice, MMP-13 expression was notably reduced. In conclusion, hUC-MSC treatment can inhibit abnormal keratinocyte proliferation, cease the cell cycle at the G1 phase, and reduce the secretion of proinflammatory cytokines in monocytes and macrophages [132]. Bone marrow-derived MSCs are currently the most commonly used MSCs in clinical practice and are the gold standard of MSC therapy. The yield of MSCs derived from adipose tissue is 500-fold higher than that from bone marrow, which is expected to be a potential source of MSCs. Gomez et al. used highly physiological condition-matched mouse medulla ossium (MO) and fat tissue (FT)-derived MSCs to investigate the therapeutic effect on imiquimod-induced mouse models. Licensed MSCs mean that the anti-inflammatory function of the MSCs has been enhanced by IFN-γ treatment combined with TNF-α and IL-1β prior to administration. Unlicensed MSCs mean no cytokine treatment before administration. Both unlicensed MO MSCs and FT MSCs reduced the total PASI score from 6.0 to 2.0. Unlicensed MO MSCs and FT MSCs upregulated the secretion of interleukin-6 while reducing epidermal thickness. The application of licensed MSCs decreased their ability to secrete IL-6; the results implied a reduction in their therapeutic potential. In conclusion, MSCs exert therapeutic effects by secreting cytokines themselves, thereby influencing the pathological process of psoriasis [133]. Ding et al. injected IFN-γ- and TNF-α-stimulated umbilical cord-derived MSCs subcutaneously into psoriasis lesions, and neutrophil infiltration was significantly reduced. They inferred that MSC-IT suppresses inflammation through the secretion of TSG-6. The results showed that the therapeutic effect of TSG-6 siRNA-transduced MSCs was lost. Moreover, their findings revealed that TSG-6 has the capacity to suppress the recruitment of neutrophils through the downregulation of CXCL1 expression. This mechanism is potentially linked to the decreased levels of STAT1 phosphorylation observed in keratinocytes [134].

Lin et al. developed an optimized acellular method for extracting ECM from fish skin, which was then mixed with metal Ru and persulfate salt to produce a photoactivated ECM bioink. The bioink can be controlled using microfluidic technology to prepare porous ECM microcarriers in situ under the catalysis of light. Under the modification of polydopamine, MSCs can adhere to these microcarriers well. The PD-L1 expression of MSCs was enhanced using lentiviral transfection. These microcarriers can prolong the survival time of MSCs in vivo. In an in vivo psoriasis model, epidermal thickening was found to be effectively inhibited, and the PASI score was reduced after microcarrier administration [135]. Wharton’s Jelly, a connective tissue that contains collagen, hyaluronic acid, and sulfate proteoglycans, is an abundant source of MSCs. Compared with MSCs derived from bone marrow and other tissues, human Wharton’s Jelly mesenchymal stem cells (hWJ-MSCs) are less mature, enabling them to proliferate more efficiently and rapidly while exhibiting lower immunogenicity. At the same time, hWJ-MSCs also have immunomodulatory and anti-inflammatory properties, which is consistent with other sources of MSCs. Carrillo et al. evaluated the potential of hWJCM, a secreted product of hWJ-MSCs, to treat psoriasis. They administered hWJCM loaded in a hyaluronic acid (HA) matrix. HWJCM-HA reduced TNF-α expression levels by 77% and decreased the PASI score from 8.5 to 2.0. It was found that hWJCM-HA had strong angiogenic potential. HWJCM-HA can reduce the degree of distortion and complexity of blood vessels and can also reduce epidermal hyperplasia and keratosis [136]. MSCs are considered multipotent cells capable of differentiating into various human tissues. Extensive clinical research has been carried out to utilize MSCs for repairing damaged tissues, particularly the heart. Researchers now recognize that MSCs produce numerous bioactive factors and possess innate immunomodulatory functions, making them a potential treatment for a variety of immune-related diseases. However, MSCs do not yield therapeutic effects for all patients. Factors such as production methods, cell viability, delivery platforms, the severity of the disease, and genetic receptivity can all influence the efficacy of MSCs. Currently, the focus lies on screening suitable MSCs for specific diseases and enhancing the properties of MSCs to improve their therapeutic potential.

### 4.2. Extracellular Vesicles

Over the past few years, small extracellular vesicles (sEVs) have been explored as cell-free immunomodulators. In fact, unmodified or engineered sEVs have been affirmed to have therapeutic potential in different types of diseases, including cancer, inflammatory lung disease, and autoimmune diseases.

The programmed cell death of protein 1 and its ligand (PD-1/PD-L1) signaling axis play an essential role in preserving immune homeostasis by suppressing both the initiation and effector stages of immune reactions. Evidence suggests that dysfunctional PD-1/PD-L1 signaling is closely associated with the development of autoimmune disorders. Augmentation of the PD-1/PD-L1 binding capacity has been demonstrated to suppress the expansion and activation of antigen-targeted T lymphocytes and B lymphocytes while concurrently curbing the secretion of inflammatory cytokines.

Recently, Xu et al. developed bone marrow-derived MSC extracellular vesicles that highly expressed PD-L1 using lentiviral transfection and puromycin screening, achieving an average diameter of 100 nm. This minimal size lowered the risk of thrombosis and vascular blockage. The researchers observed that MSC-sEV-PD-L1 treatment alleviated skin redness and scaling and significantly reduced acanthosis, parakeratosis, and epidermal thickening. Additionally, vasodilation and immune cells were diminished from the skin [137].

Rodrigues et al. introduced umbilical cord-derived sEVs (UCB-sEVs). These vesicles induced M2 macrophage polarization, which subsequently influenced skin fibroblasts, reducing their reactivity to inflammatory signals. UCB-sEVs reduced the number of CD8+ T cells infiltrated while increasing Treg cell populations. Furthermore, UCB-sEV treatment significantly lowered the level of inflammatory molecules, such as interferon and chemokine, as well as antimicrobial peptides. UCB-sEV treatment also promotes a transformation from T helper cells to regulatory T cell phenotypes, restoring local immune balance and demonstrating the potential for psoriasis treatment [138].

**Figure 4:**
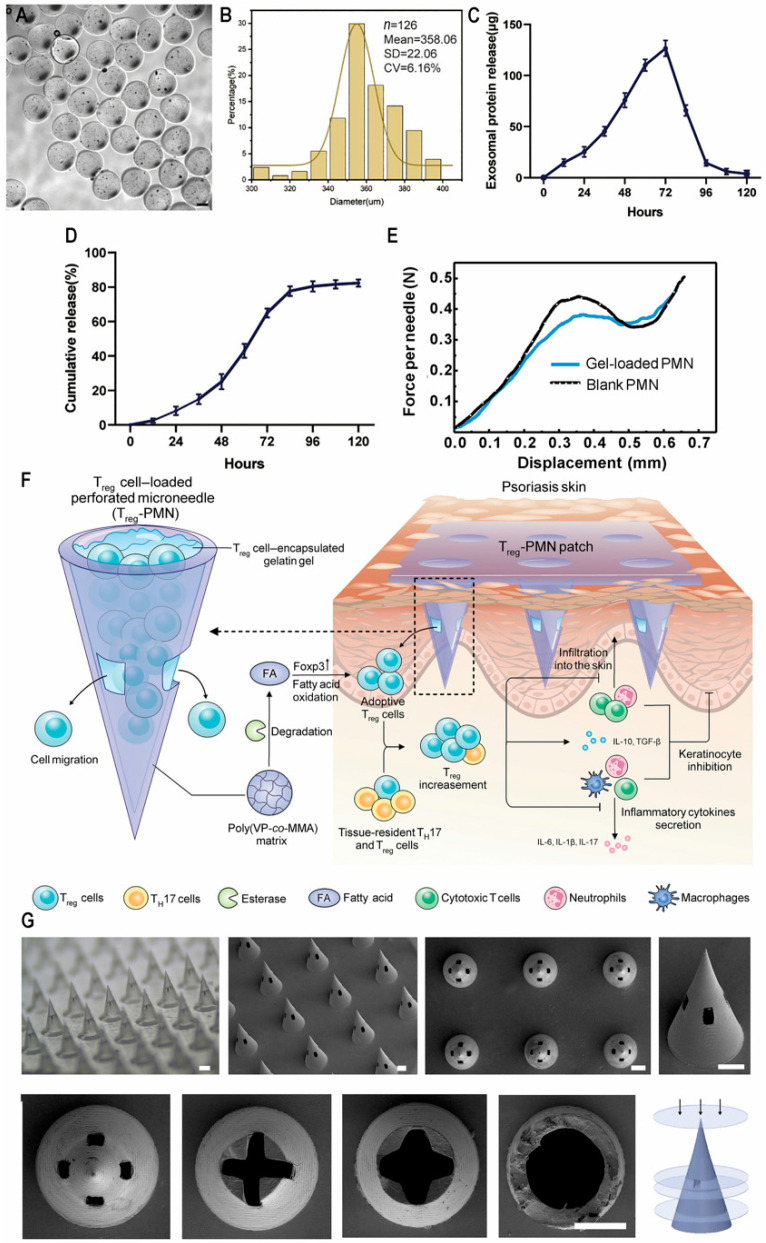
(**A**) Optical photograph of CA-EV-M. Scale bar: 200 μm. (**B**) The size distribution of CA-EV-M was shown in two forms: histogram and frequency distribution curve. (**C**) Quantitative results of protein in vitro released from CA-EV-M over 5 days. (**D**) Quantified cumulative protein release of CA-EV-M. Reproduced from Ref. [140]. (**E**) Mechanical properties of the PMNs. (**F**) Psoriasis therapeutic mechanism of PMNs. (**G**) Microstructure of the PMNs via SEM (scale bars: 300 μm, 200 μm, 300 μm, 200 μm, and 300 μm, respectively, from left to right). The arrows indicate the direction of observation. Reproduced with permission from Ref. [143].

Jiang et al. proposed a new therapeutic strategy for psoriasis using JPH203-loaded extracellular vesicles derived from UVB-irradiated keratinocytes (J@EV). JPH203, a specific LAT1 inhibitor, blocks leucine uptake to suppress mTOR signaling. J@EV exhibited enhanced uptake in inflamed HaCaT cells, with 250% upregulation in fluorescence intensity compared with normal cells after one hour of incubation. J@EV reduced epidermal thickening by 77% and decreased the Th17 cell ratio in the spleen from 19.6% to 4.25% compared with the IMQ group. J@EV also suppressed the phosphorylation of mTOR, reducing the p-mTOR/mTOR ratio by 60%. In summary, J@EV demonstrated potent therapeutic efficacy in psoriasis by targeting the vicious “Inflammation-Th17-Keratinocytes” cycle, with significant reductions in epidermal thickness, PASI scores, and immune cell activation while maintaining good biosafety [139]. At present, researchers are focusing not only on the disturbance of the immune system in psoriasis but also on the disturbance of the skin microbiome, which plays an increasingly prominent role in psoriasis. Bacterial extracellular vesicles play an important regulatory role in host–microbiome interactions. Xu et al. extracted extracellular vesicles (CA-EVs) derived from *Cutibacterium acnes* (*C. acnes*), embedded them in gelatin methacrylate (CA-EV-G), and prepared hydrogel microsBpheres (CA-EV-M) with an average diameter of 358.06 ± 22.06 μm (Figure 4A,B). CA-EV-M presented the sustained release of CA-EVs, with about 80% of the total protein content within 4 days (Figure 4C,D). The epidermal thickness decreased from 120 μm in the IMQ group to 60 μm in the CA-EV-M group. TEWL was reduced by 50% in the CA-EV-M group compared with the IMQ group. The EVs of *Staphylococcus epidermidis* play a role in regulating the immune environment by interacting with the host skin [140]. Chavez et al. extracted extracellular vesicles (EVs) from two different strains: a symbiotic strain, designated ATCC12228EVs, and a clinically isolated strain, referred to as 983EVs. Both types of extracellular vesicles (EVs) were observed to increase IL-6 expression in HaCaT cells. However, 983EVs caused a more pronounced upregulation of several proinflammatory biological macromolecules, including VEGF-A, compared with ATCC12228EVs. Additionally, ATCC12228EVs were more effective than 983EVs in alleviating disease characteristics of IMQ-induced psoriatic skin symptoms while also reducing the expression of inflammatory cytokines mentioned before; moreover, the expression of IL-36 receptor antagonists increased. A proteomic analysis revealed the distinction of protein expression between the two sources of EVs. In ATCC12228EVs, some proteins may participate in the repair process of psoriatic lesions, such as glutamate dehydrogenase and ornithine carbamoyltransferase. In summary, their study showed that the commensal ATCC12228EVs had better psoriasis efficacy compared with the clinically derived 983EVs [141]. EVs facilitate intercellular communication and are abundant in source, allowing for specific modifications as needed. They are also utilized for diagnosing and monitoring disease progression, making them a highly promising therapeutic tool. Clinical trials involving EVs date back to 1999, and in recent years, the number of such trials has grown exponentially. Currently, most clinical trials involving EVs focus on diagnostics, with EVs derived from biological fluids becoming the gold standard for diagnosing certain diseases, particularly cancer. In contrast, EV-based therapies are primarily targeted at respiratory diseases, a focus that has been bolstered by the recent pandemic. However, the progress of EV-based treatments is hindered by limitations in isolation technologies and the inability to fully validate the functional subtypes within EVs from the same source. In summary, the clinical translation of EVs remains challenging [142].

### 4.3. Adoptive Cell Therapy

Regulatory T cells hold promise for anti-inflammatory therapy via adoptive cell transfer; however, in the context of skin autoimmune diseases, the systemic administration of these cells frequently fails to achieve adequate tissue-specific targeting and accumulation. Additionally, the instability and plasticity of Treg cells may result in phenotypic changes and functional loss. Zhang et al. designed perforated microneedles (PMNs) that exhibit excellent mechanical strength (Figure 4E) and feature a large encapsulation space to promote cell viability. Additionally, these microneedles incorporate an adjustable channel system that enhances cell penetration, making them suitable for topical psoriasis treatment. In addition, the microneedle substrate can be degraded by enzymes to release fatty acids in the inflammatory region and enhance the inhibition function of Treg through metabolic intervention mediated by fatty acid oxidation (FAO) (Figure 4F,G). In mouse models of imiquimod-induced psoriasis, PMN-delivered regulatory T cells demonstrated multi-faceted therapeutic effects, including a marked reduction in leukocyte infiltration (with a notable attenuation of neutrophil migration), concomitant inhibition of proinflammatory cytokine production, upregulation of anti-inflammatory mediators, and ultimate restoration of dermal immune equilibrium [143].

Dantas and colleagues established an inducible transgenic murine model (ihTNFtg) expressing human TNF, which manifested an arthritis-resembling psoriatic disease following doxycycline administration. These genetically modified mice exhibited cutaneous abnormalities marked by keratinocyte overproliferation and dysregulated activation, accompanied by elevated proinflammatory cytokine levels with concurrent infiltration of Th1 lymphocytes and regulatory T cells. Notably, pathological macrophage accumulation in lesional skin tissue suggested a psoriasiform phenotype. Experimental evidence demonstrated that Treg deficiency in wild-type mice exacerbated both cutaneous inflammation and macrophage recruitment, whereas adoptive transfer of FOXP3+ cells effectively attenuated psoriatic manifestations and myeloid cell infiltration. These findings collectively indicate that regulatory T lymphocytes exert inhibitory effects on macrophage-mediated proinflammatory responses, identifying macrophages as principal immunopathological effectors in TNF-driven psoriatic pathogenesis [144].

## 5. Discussion

At present, the drugs for treating psoriasis are mostly topical preparations and monoclonal antibodies. Different therapies are available for psoriasis of varying severity. Regarding small-molecule drugs, their advantages lie in convenient administration through oral and topical forms, low cost, easy storage and transportation, and simple and stable preparation technology. On the other hand, the advantages of biological agents are strong specificity, fewer adverse reactions, better potency in the treatment of psoriasis in the severe type, and lower dosing frequency (Table 1).

The existing delivery systems still have certain limitations: 1. The thickened epidermis limits the transdermal efficiency and therapeutic effect of topical preparations. 2. Toxicity can be inherent in small-molecule drugs, and adverse reactions may be caused by off-target effects of biological drugs. 3. Frequent dosing affects patient compliance. 4. The production technology of biological drugs is still in its infancy. Nanocarriers can optimize the treatment of psoriasis. Firstly, functionalized nanocarriers can generate good targeting effects and improve transdermal efficiency and retention ability. Secondly, new delivery systems can also increase the bioavailability of psoriasis drugs, improve therapeutic effects, and reduce adverse reactions. Moreover, nanocarriers make it possible to simultaneously deliver two or more therapeutic agents, providing a platform for the synergistic effect of drugs.

The realization of the clinical translation of new delivery strategies should be pursued (Table 2). The reproducibility (particle size) of nanocarriers between batches is poor. Nanocarriers are rapidly cleared in biological fluids; they accumulate in specific tissues and organs (such as the liver and kidney) in large quantities and cause toxicity. There is a lack of unified regulations and standards regarding the development and administration of nanocarriers. Compared with traditional drugs, the development cost of nanocarriers is higher. These are factors that restrict the clinical transformation of nanocarriers. In the future, the technical means for drug manufacturing will become more diverse. The discovery of new drug targets and the exploration of pharmacological effects will provide new ideas for the optimization of nanocarriers. Multi-disciplinary and multi-field cooperation will also contribute to the breakthrough of drug delivery technology. However, during this process, ethical and moral concerns should be noted. High production costs and intellectual property barriers may limit access to treatments for low-income populations. Patients must understand risks, such as unknown immune reactions to nanomaterials. The regulatory framework must balance innovation and patient safety and ensure fair allocation and transparency of clinical trials.

In the future, with the progress of medical services, personalized nanomedicines will gradually become a trend. More intelligent delivery carriers will also emerge, such as stimulus-responsive carriers. In addition, carrier design assisted by artificial intelligence has also received increasing attention.

## 6. Conclusions

Psoriasis is a prevalent skin disease with no available therapy that can completely cure and prevent relapse thus far. Novel drug delivery systems of traditional small-molecule medicines and the emergence of biological therapies make completely removing skin lesions and curing psoriasis possible. This comprehensive analysis examines cutting-edge therapeutic platforms for small-molecule pharmaceuticals, which have demonstrated efficacy in optimizing dermal permeation, engineering controlled-release kinetics, elevating pharmacokinetic performance, minimizing adverse reactions, and augmenting therapeutic adherence. The discourse further elucidates the revolutionary paradigm shift in biologics development, encompassing the evolutionary trajectory of psoriasis-targeted monoclonal antibody engineering and next-generation biomacromolecule transport modalities (including protein-based therapeutics and nucleic acid vectors) alongside emerging adoptive cell therapy strategies. These innovative therapeutic architectures aim to revolutionize psoriasis management through precision-targeted molecular intervention and cellular reprogramming paradigms. However, most of these studies remain in animal experiments currently, and much work remains to be carried out before clinical application. In the future, the emergence of new delivery systems and advancements in artificial intelligence will drive progress in the treatment of psoriasis. Comprehensive research is required for the successful translation of innovative psoriasis therapy. However, the current research into the development of new psoriasis therapy has profound significance, and successful clinical translation and application are still the goals of researchers.

## Figures and Tables

**Table 1:** Comparative table for different drug delivery systems.

Drug	Delivery System	Administration Route	Characterization	Improvement	Reference
Dexamethasone	Hydrogel	Topical	Temperature-responsive property and modified prodrug	Immune microenvironment-responsive and lower dosage	[29]
	Microneedles	Topical	Liposome-loaded	High permeation efficiency	[34]
	Gold nanorods	Topical	Near-infrared laser-assisted	High skin permeation, epidermis retention, and lower risk of systemic side effects	[35]
Methotrexate	Hydrogel	Topical	Photodynamic assisted therapy	Noninvasive manner and high permeation efficiency	[48]
	Microneedles	Topical	Modified prodrug and ROS responsiveness	High targeting efficiency	[60]
	Niosomes (non-ionic surfactant vesicles)	Topical	Co-delivery of MTX and niacinamide	Improved skin permeation and retention	[63]
Tazarotene	PLGA nanoparticles	Topical	Follicular delivery	Controlled and extended-release	[79]
	Solid lipid nanoparticles	Topical	Solubilization of poorly soluble drugs	Improved viscoelastic properties	[82]
Calcipotriol	Microneedles	Topical	Soluble microneedles	Good biocompatibility	[85]
	Micelles	Topical	Modified prodrug and ROS responsiveness	Reduced off-target efficiency	[88]
	Nanoemulsion	Topical	Natural components	Enhanced skin permeation	[89]
siRNA	Framework nucleic acid	Topical	siRNA targeting NF-κB	Excellent transdermal efficiency	[102]
	Polypeptide platforms	Intravenous injection (i.v.)	siRNA targeting TNF-α	High macrophage targeting mediated by the protein corona	[103]
	Ionic liquids	Topical	siRNA targeting Fn14	High knockdown efficiency	[107]
Cas9 ribonucleoprotein	Microneedles	Topical	NLRP3 inflammasome elimination	Promoted indel efficiency	[98]
	Polymer nanoparticles	Subcutaneous injection (s.c.)	Co-delivery of lipoic acid and RNPs	High cellular internalization	[99]
MSCs	/	i.v.	Derived from bone marrow and adipose tissue	Healing response acceleration and severity alleviation	[134]
	/	i.v.	Derived from umbilical cord	Proinflammatory cytokine reduction and blocking of keratinocyte proliferation	[132]

**Table 2:** Ongoing clinical trials for psoriasis treatment.

Drug	Type	Formulation	Physiological Target	Phase	No.
VTP-43742	Small molecule	Oral tablet	Retinoic acid receptor-related orphan receptor gamma t (RORγt)	Phase II	NCT05153148
Roflumilast Cream (ZORYVE^®^)	PDE4 inhibitor	Topical cream	Phosphodiesterase-4 (PDE4) in skin cells	Phase III	NCT05028582
Upadacitinib (RINVOQ^®^)	JAK1 inhibitor	Oral tablet	Janus kinase 1 (JAK1)	Phase III	NCT03569293
Deucravacitinib (BMS-986165)	TYK2 inhibitor	Oral tablet	Tyrosine kinase 2 (TYK2) in JAK-STAT pathway	Phase III	NCT05650827
Sonelokimab (M1095)	Nanobody (anti-IL-23)	Subcutaneous injection	IL-23/IL-17 pathway	Phase II/III	NCT05643590
Spesolimab (BI 655130)	Monoclonal antibody	Intravenous infusion	IL-36 receptor (for pustular psoriasis)	Phase III	NCT05799801
Bimekizumab	Dual IL-17A/IL-17F inhibitor	s.c.	IL-17A and IL-17F cytokines	Phase IV	NCT06026900
Tapinarof (VTAMA^®^)	Aryl hydrocarbon receptor (AhR) modulator	Topical cream	AhR pathway to reduce inflammation	Phase IV	NCT05604960
Exagamglogene autotemcel (exa-cel)	CRISPR-Cas9 gene-edited therapy	Intravenous infusion	Gene correction in hematopoietic stem cells (early-phase exploration for autoimmune diseases)	Preclinical/Phase I	/
Tofacitinib-loaded nanoparticles	JAK inhibitor + nanotechnology	Topical gel	Localized JAK/STAT pathway inhibition	Phase I/II	NCT05462071
